# The Crowded Sea: Incorporating Multiple Marine Activities in Conservation Plans Can Significantly Alter Spatial Priorities

**DOI:** 10.1371/journal.pone.0104489

**Published:** 2014-08-07

**Authors:** Tessa Mazor, Hugh P. Possingham, Dori Edelist, Eran Brokovich, Salit Kark

**Affiliations:** 1 ARC Centre of Excellence for Environmental Decisions, School of Biological Sciences, The University of Queensland, Brisbane, Queensland, Australia; 2 Grand Challenges in Ecosystems and the Environment, Silwood Park, Imperial College, London, United Kingdom; 3 Leon Recanati Institute for Maritime Studies, Department of Maritime Civilizations, University of Haifa, Mount Carmel, Haifa, Israel; 4 Department of Geography, The Hebrew University of Jerusalem, Mount Scopus, Jerusalem, Israel; UC Santa Cruz Department of Ecology and Evolutionary Biology, United States of America

## Abstract

Successful implementation of marine conservation plans is largely inhibited by inadequate consideration of the broader social and economic context within which conservation operates. Marine waters and their biodiversity are shared by a host of stakeholders, such as commercial fishers, recreational users and offshore developers. Hence, to improve implementation success of conservation plans, we must incorporate other marine activities while explicitly examining trade-offs that may be required. In this study, we test how the inclusion of multiple marine activities can shape conservation plans. We used the entire Mediterranean territorial waters of Israel as a case study to compare four planning scenarios with increasing levels of complexity, where additional zones, threats and activities were added (e.g., commercial fisheries, hydrocarbon exploration interests, aquaculture, and shipping lanes). We applied the marine zoning decision support tool Marxan to each planning scenario and tested a) the ability of each scenario to reach biodiversity targets, b) the change in opportunity cost and c) the alteration of spatial conservation priorities. We found that by including increasing numbers of marine activities and zones in the planning process, greater compromises are required to reach conservation objectives. Complex plans with more activities incurred greater opportunity cost and did not reach biodiversity targets as easily as simplified plans with less marine activities. We discovered that including hydrocarbon data in the planning process significantly alters spatial priorities. For the territorial waters of Israel we found that in order to protect at least 10% of the range of 166 marine biodiversity features there would be a loss of ∼15% of annual commercial fishery revenue and ∼5% of prospective hydrocarbon revenue. This case study follows an illustrated framework for adopting a transparent systematic process to balance biodiversity goals and economic considerations within a country's territorial waters.

## Introduction

Implementing marine conservation plans is a major challenge. Plans that determine priority areas for conservation are often based solely on biological and ecological information [1] One of the main factors inhibiting the uptake of marine conservation plans by decision makers is inadequate consideration of the broader social and economic context within which conservation operates [2]–[4]. Marine waters and their biodiversity are shared by a host of stakeholders and interest groups, such as commercial fishers, recreational users and offshore developers [5]. Inclusion of the activities of these multiple marine users within conservation plans is critical for achieving plans which are realistic and achievable in the real world, thereby moving from paper to action [2].

Conservation planners must try to explicitly consider other marine activities within conservation plans, to ensure no time is wasted over trying to conserve areas essential for other uses [6]. Competition for ocean space is becoming increasing intensified as resource extraction and developments are expanding to include the marine realm [7]. Offshore activities such as commercial fishing, aquaculture facilities, sand mining, desalination plants, offshore wind farms and offshore power plants, provide countries with substantial economic gains [5]. Currently, hydrocarbon operations are one of the largest economic stakeholders in the sea [8], and provide countries with huge potential and realized monetary benefits, and are expected to increase economic and political independence [9], [10]. However, incorporation of such economic activities is often absent from marine conservation planning literature. Despite the little willingness for countries to protect marine areas that are deemed economically important [11], excluding other marine activities in conservation planning means we may not be able to design a marine reserve network that is representative or economically viable [12].

Disregarding other marine activities in marine conservation planning may also mean that anthropogenic threats to biodiversity are being ignored. When planning marine reserves that aim to reap sustainable long-term benefits it is important to examine the threats to biodiversity of the system that could impair this goal. However, reserve planning should not be solely based upon threat data [13]. Examples of threats to biodiversity for consideration in reserve planning include: shipping lanes which pose a collision risk to marine mammals [14], trawlers and demersal longliners which are damaging to benthic environments and responsible for the majority of annual sea turtles deaths via by-catch [15], and marine energy installations which have been linked to habitat loss, noise pollution and invasive species [16]. In some cases marine users have made changes or modifications, such as altering the path of shipping lanes for cetaceans [17]. However, in cases where compromises cannot be met, conservation planners must be able to incorporate the potential threats to biodiversity into the planning process.

A common misconception is that marine zoning itself is a conservation planning tool. Marine zoning is the allocation of particular activities to specified marine areas [5], [18]. This practice can help reduce user conflict by separating incompatible activities [7], [18], [19]. Several countries have stepped up to implement zoning strategies for their waters, the largest and perhaps most successful example of marine zoning is the Great Barrier Reef Marine Park off the coast of Queensland, Australia [20], [21]. More recent zoning efforts occurring around the globe include the United Kingdom Irish Sea Pilot [22], the Belgian Exclusive Economic Zone [11], the waters of Norway [18], Australia's entire commonwealth waters [23] and the zoning of China's territorial sea [24]. However, key elements are often missing from some zoning plans to ensure biodiversity goals are met. For marine zoning to be used as an appropriate method or tool for protecting marine biodiversity it must enable an explicit consideration of the trade-off between biodiversity and socio-economic objectives [25]. Furthermore, zoning plans need to ensure that the zoning system provides protection that is representative of as many biodiversity features as possible [12], [25].

The concept of including other activities within marine conservation planning is slowly emerging. Unlike marine spatial planning (MSP) which aims to plan water spaces to meet objectives of multiple marine users and stakeholders, [19], [26], marine conservation planning (MCP) is centred on one primary goal - achieving biodiversity protection [18]. Recently, several systematic conservation plans in the marine realm have focused on a hybrid approach; reaching conservation objectives while also minimizing the opportunity cost to fishery stakeholders [25], [27], [28]. However, only some of these plans have been expanded to other social and economic contexts (e.g., [3], [29]). Facilitating the inclusion of other activities into marine conservation planning is the emerging development of zoning software that enables multiple objectives to be considered (e.g., Marxan with Zones [27]). Up to now there has been little application of these new tools to address the complexity of marine conservation planning at regional scales or an entire country scale. As many countries around the globe aim to implement conservation measures by zoning their waters [18], it is important to develop an explicit zoning process which integrates the current spatial occupancy of other marine activities and where possible their economic objectives. The inclusion of other marine uses in marine conservation planning means that we need to carefully consider the trade-offs that underpin the resulting conservation plans and ensure that biodiversity goals are adequately achieved.

**Figure 1:**
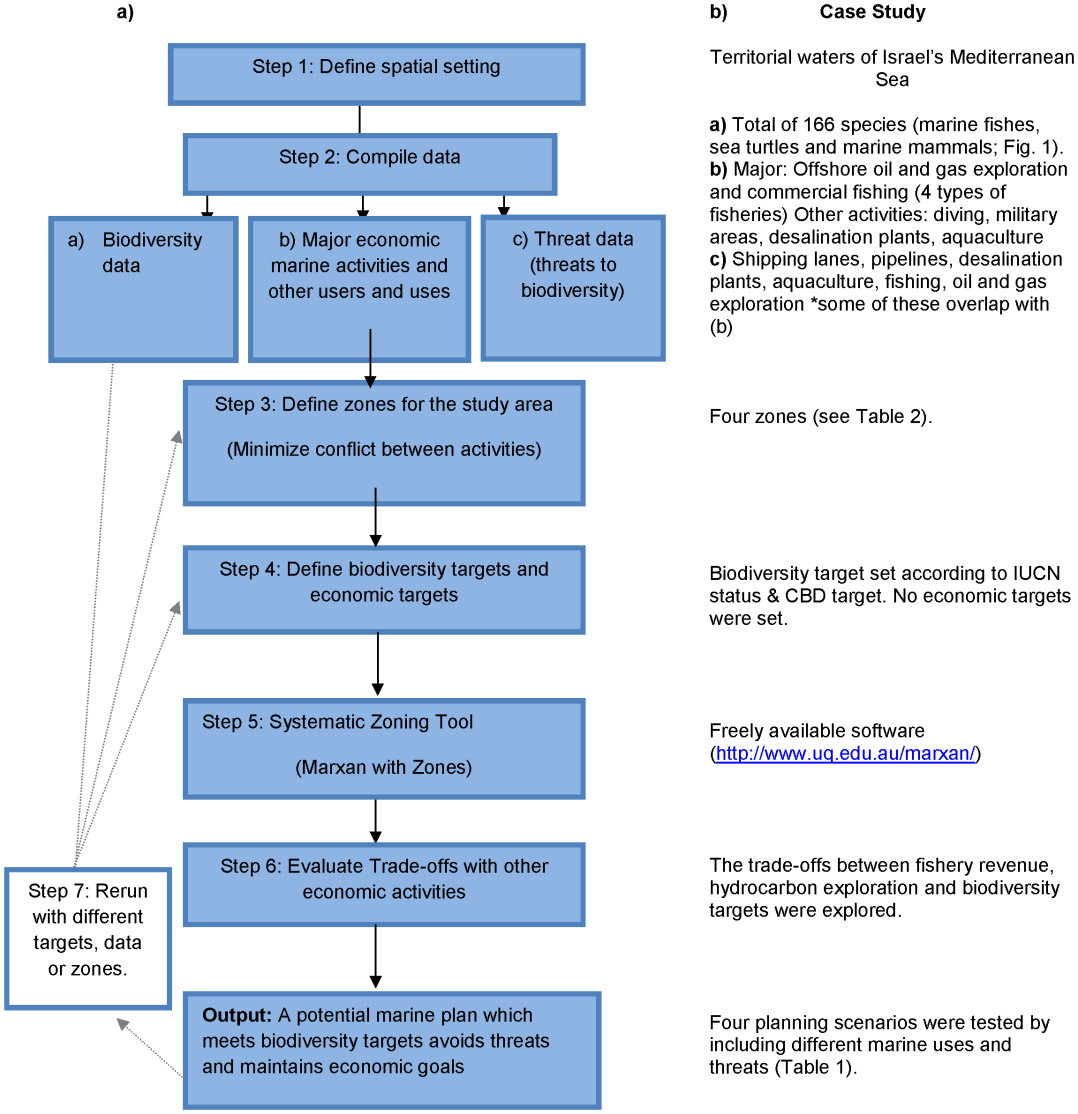
Proposed framework for incorporating multiple activities and threats into marine conservation planning. These show the steps followed in the case study presented in this paper that encompasses Israel's entire Mediterranean territorial waters.

In this study we follow a framework (Fig. 1) using a systematic approach for zoning territorial waters to achieve the protection of marine biodiversity in the face of multiple anthropogenic threats and economic activities. Within this context, we aim to test how increased complexity (by the inclusion of zones, multiple activities and economic factors) in marine conservation planning alters: a) the ability to reach biodiversity targets, b) the opportunity cost, and c) the spatial conservation priorities. Furthermore, we aim to examine the explicit incorporation of prospective hydrocarbon extraction into marine conservation planning [8].

## Methods

Here our methods follow the steps outlined in Figure 1.

### Spatial setting and study area

As a case study, we examined Israel's complete Mediterranean territorial waters. Israel is located in the eastern Mediterranean Sea and has relatively small territorial waters (∼4200 km^2^) compared with other coastal countries around the world. Currently, it faces rapid exploitation of its marine resources and aims to expand its protection of marine biodiversity [30]. Israel's Mediterranean Sea territorial waters are defined by the National Planning Authority of Israel and are used by The Israel Nature and Parks Authority (NPA) for marine reserve planning. The territorial waters of Israel's Mediterranean Sea spreads along a coastline ∼190 km long, and extends outwards for 12 nautical miles from the coast to a depth of ∼1000 m, covering an area of ∼4200 km^2^ [30]. For our analyses, we divided this study area into 1×1 km planning units, resulting in a total of 4,205 planning units.

### Compiling biodiversity features

**Figure 2:**
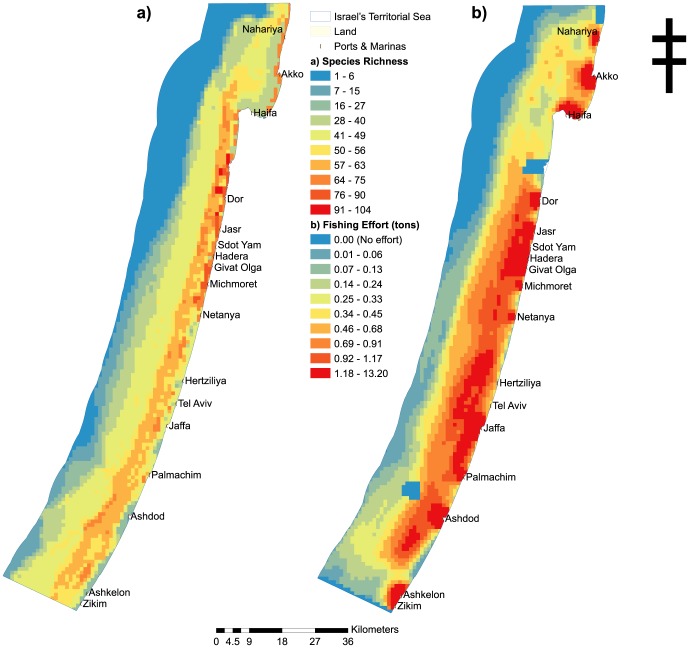
Biodiversity features and fishing effort in Israel's Mediterranean Sea territorial waters; a) species richness of 166 biodiversity features (species and geomorphologic features), b) combined fishing effort (entangling nets, longliners, purse seiners and trawlers), where the blue areas (no effort) are restricted fishing areas; marine reserves, military areas and aquaculture.

In order to select marine areas which will fulfil a representative reserve network where all types of biodiversity are protected we compiled available distribution data of Israel's Mediterranean territorial waters of biotic and abiotic features. These included 166 biodiversity features, comprising of vertebrate marine species (153 fishes, 2 turtles, 1 cetacean), and 10 geomorphologic features (Fig. 2a; see Table S1 in File S1. for a list of species and features included in this study).

#### Marine species distribution data

We compiled data from currently available published studies on native cartilaginous and bony fishes whose distribution lies within Israel's Mediterranean waters [31]–[38]. All native (non-alien) fish species (153 species) present in these publications were included in our study. We digitized the documented depth ranges of these native fish species using ArcGIS ([39]; Fig. S1 in File S1.; Table S2 in File S1.) and sea floor bathymetry [40], following methods in Tognelli et al. [41] and Clark & Tittensor [42]. We derived the distributions via a number of sources; locations and depth ranges from the above eight studies, data from the Hebrew University of Jerusalem Fish Collection (accessed 2012), ranges as documented in Golani et al. [43], and by expert opinion (for further details see File S2).

The distribution of sea turtles within Israel's marine waters has not been well documented and their preferred feeding, foraging and mating areas are currently poorly known. Therefore, we used the locations of established nesting sites (within [44] in Israel for both the green (*Chelonia mydas*) and loggerhead (*Caretta caretta*) sea turtle species. The targeted nesting habitats for protection in this study were chosen as planning units adjacent to nesting beaches with over 20 nest counts (from 1993–2011) and a persistence of more than five years of nesting at a particular site, in accordance with expert opinion from rangers and scientists at Israel's Nature and Parks Authority and Sea Turtle Rescue Centre.

We included the distribution of the common bottlenose dolphin (*Tursiops truncatus*), the most common cetacean species in Israel's territorial waters. Other cetacean species exist in Israel's waters but not enough observational data exists to determine priority habitats for these species. The common bottlenose dolphin has been sighted throughout Israel's territorial waters, therefore to better direct our conservation efforts we have considered important habitat areas as the species distribution. Scheinin [45], identified three core areas for feeding and foraging, an area at a depth of 40–50 m near Ashkelon, an area at a depth of 30–60 m between Ashdod and Palmachim beaches and another area off the coast of Netanya at a depth of 90–120 m. These three core habitat areas cover in total 213.64 km^2^ (for additional information see File S2.).

#### Geomorphological features

In order to represent different types of marine habitats we included geomorphologic features to serve as surrogate “biodiversity features”. We used ten geomorphologic features within Israel territorial waters that were mapped (in 2008) and provided by The Israel Nature and Parks Authority. These features include: shallow rocks, kurkar (calcareous aeolianite) ridges, kurkar bustan, deep kurkar ridges, continental shelf silt, continental shelf sand, continental ridges, large canyons, continental slope and canyons, deep sea [46].

#### Setting biodiversity targets

Biodiversity targets were set to protect a percentage of the species distribution according to its level of global threat based on the IUCN red list criteria ([46], Table S1 in File S1.) and current range size. We set a 10% target for species that were listed “Least Concern” by the IUCN and all other fish species that have not been evaluated by IUCN. This target was increased to 15% for species listed “Vulnerable” by the IUCN [47] and to 20% for species listed “Endangered” by the by the IUCN. Species listed “Endangered” that had a distribution of less than 1% of the study area were given a target of 50%. For the geomorphological features, we set a target to protect 5% of all features and those that are represented by an area less than 1% of Israel's territorial waters were given a 10% target. We also set a constraint that at least 5% of the distribution of all species and features must be placed within the no-take zone (Conservation Zone), meaning that the rest of the biodiversity target could be fulfilled in other zones. While our target setting approach does not consider whether the target is adequate at conserving the species or maintaining population viability, it aims to address the IUCN criteria that defines the risk of species extinction as applied in Kark et al. [48] and Lieberknecht et al [49]. To test the sensitivity of our results we also used a 10% target for each species and a 5% target for each geomorphologic feature.

### Incorporating economic activities in the sea

We included the two major economic activities in the Mediterranean waters of Israel (commercial fishing and hydrocarbon operations) in the conservation planning exercise. While there are other localized marine activities and features (addressed below; Table 1) commercial fishing and hydrocarbon operations are activities that span across Israel's territorial waters and rely on resource extraction. Thus, we focused on these activities which are likely to be the main source of opportunity cost incurred when implementing marine protected areas and zones. We translated these activities into opportunity cost layers for use within Marxan. Opportunity cost in this study was defined as the value of forgone economic activities (commercial fishing and hydrocarbon operations) when a particular area (planning unit) is made into a protected area that excludes these economic activities. As spatial opportunity cost data were unavailable for these activities, we developed surrogates to represent the annual revenue (approximation of annual opportunity cost) of each economic activity within our 1 km^2^ planning units. Here we used annual values to reflect the relative opportunity cost differences across the territorial waters of Israel. We used the most current available data for Israel's territorial waters for each of these activities, specifically the year 2009 for commercial fisheries and year 2012 for hydrocarbon operations. The minimal fluctuation of Israel's annual commercial fishing catch and value over the last few years suggests that the available data of these activities is relatively comparable [50].

**Table 1:** Four zones for Israel's territorial waters that restrict and permit different activities.

Activities	Zones			
	Conservation Zone “No-Take”	Benthic Protection Zone	Exploration Zone	Economic Zone “General use”
Trawling	X	X	X	✓
Purse Seiners	X	✓	X	✓
Gillnetting	X	✓	X	✓
Long liners	X	✓	X	✓
Oil and Gas Exploration	X	X	✓	✓
**Additional threats and marine activities**				
Aquaculture	X	X	X	✓
Current protected areas^1^	✓	X	X	X
Desalination plants	X	X	X	✓
Diving	✓	✓	✓	✓
Military areas	✓	✓	✓	✓
Pipelines	X	X	✓	✓
Safety area^2^	X	X	X	X
Shipping lanes	X	✓	X	✓

1 Rosh HaNikra

2 Mari B Platform

Additional threats and marine activities (listed below) in Israel's territorial waters have been locked to particular zones as per the four scenarios.

A “✓” in the column means that this activity was permitted in this zone, where an “x” it is prohibited.

#### Opportunity cost of commercial fisheries

We developed surrogate opportunity cost layers of commercial fishing by spatially mapping fishing effort for the four major commercial fishing gears used in Israel; entangling nets, longliners, purse seiners and trawlers (see Fig. S2 in File S1; Fig. S3 in File S1; File S2. for detailed methods). We derived effort maps by equations which assume effort is proportional to the number of fishing vessels at each port for each gear type and effort decreases exponentially with distance from port (methods described in Mazor et al. [51]). For each gear type we used expert opinion (total of 25 experts) to refine our effort layers. We did this by constraining our effort layer by the maximum depth that each fishing gear is used and incorporating weightings over habitats and areas that are targeted by particular gear types. For entangling nets we constrained our effort layer by a depth of 50 m (maximum depth that entangling nets are used in Israel's as confirmed by 15 entangling net fishers in Israel). For longliners, fishing effort was weighted by both distance from port and rocky habitat (targeted fishing areas) and confined to 50 m depth (confirmed by 6 longline fishers in Israel; Fig. S3 in File S1.). For purse seiners, effort was weighted across two distinct areas in the north and south at a depth between 10–50 m as determined by expert opinion (6 purse seine fishers; Fig. S2 in File S1.). Trawling effort was based on data collected from on-board GPS devices by Edelist [38] between the years 2009–2011 and trawling data from Israel's Department of Fisheries and Aquaculture ([52]; Fig. S2 in File S1.) Using these effort maps we created surrogate opportunity cost layers by overlaying the annual revenue (year 2009) reported by Edelist et al. [50] for each fishing gear type, thereby assigning monetary values to each planning unit for each fishing gear type (Fig. 2b).

#### Opportunity cost of hydrocarbon operations

Spatial data identifying offshore oil and gas operations and leased and licensed marine extraction areas was provided by Israel's Ministry of Interior from the National Master Plan of Israel (Tama 34b). Areas of Israel's Mediterranean waters are licensed to several oil and gas companies (e.g., Noble Energy, Shemen, Delek) for hydrocarbon exploration for a period of seven years [53]. If economically viable resources are found within these licensed areas they can then be leased by energy companies with a fifty year production permit. Unexplored “blank” areas will be temporarily left aside as Israel is trying to limit exploration into these new areas. The licensed areas that were not explored will be recycled if there is no exploration in them.

As no reliable data sources were available for a total estimation of the value of Israel's offshore oil and gas reserves we performed calculations using data from Israel's Ministry of Energy and Water Resources [53], [54] (Table S3 in File S1.) and converted these estimated reserve quantities into monetary values. We multiplied the annual average international market price of oil (NIS per barrel = 404.52 in 2012; World Bank http://www.worldbank.org/) and natural gas (NIS per thousands of cubic meters = 399.33; International Monetary Fund http://www.imf.org/external/index.htm) with Israel's estimated reserve volumes. These calculations resulted in a static estimate (year 2012 values) of the value of Israel's oil and gas reserves (not including extraction cost), but we realize that prices will fluctuate annually and are expected to reach higher values in the future, thus our calculated values are expected to be an under estimate (unless estimated reservoirs will be smaller than predicted). We have estimated the value of Israel's offshore oil and gas reserves at US$ ∼324 billion (∼1,250 billion NIS; Table S3 in File S1.), with 15% of this amount retrieved from the territorial waters (US$ 50 billion). Our resulting equation gives a greater weighting to the opportunity cost of leased areas (known sources of oil and gas; α = 1) compared to licensed areas (half weighting α = 0.5):
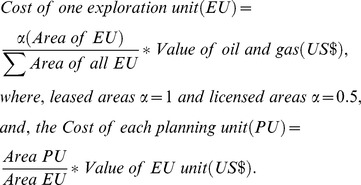



#### Considering additional marine activities in conservation planning

**Figure 3:**
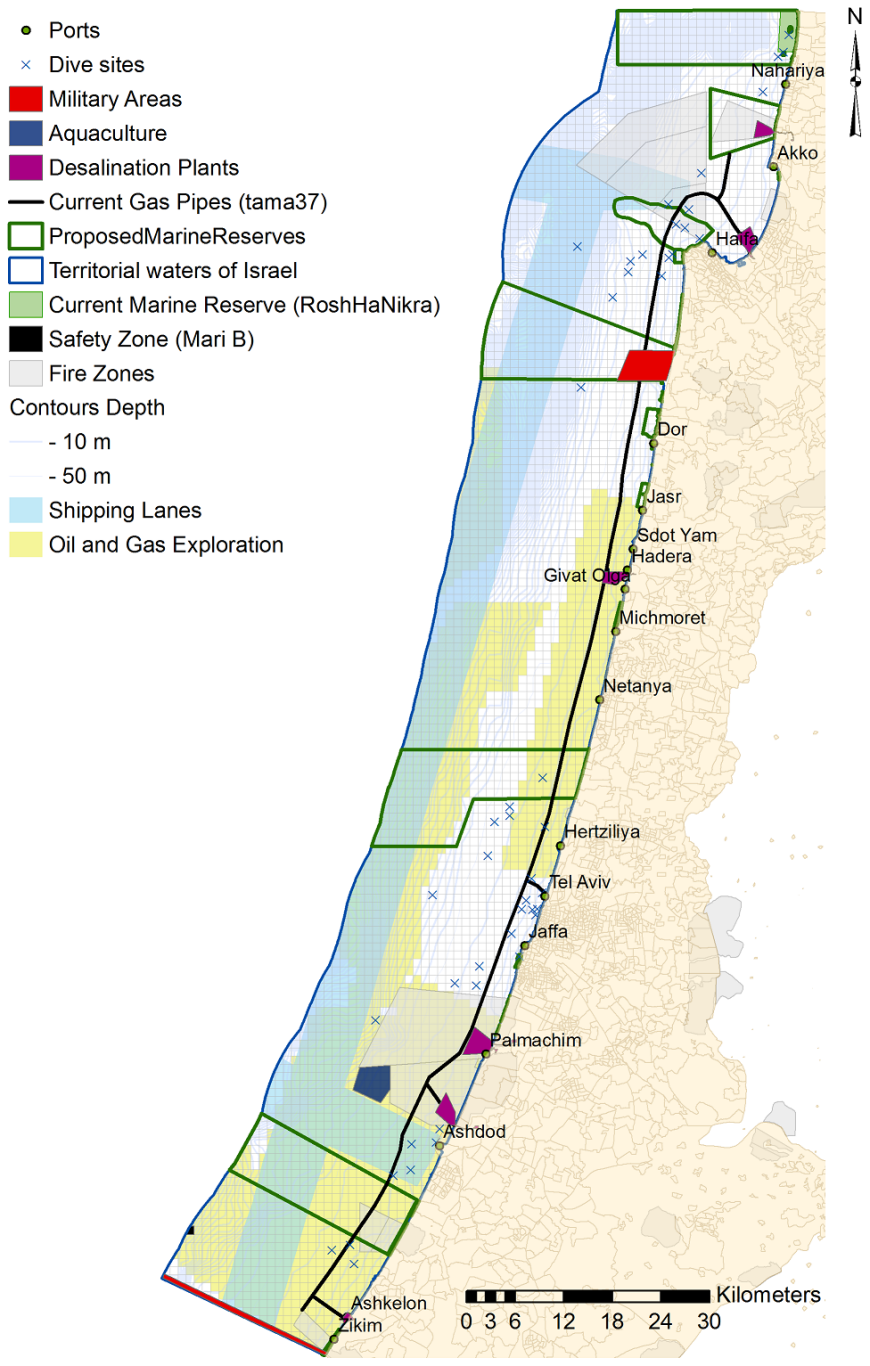
A map of the activities of Israel's Mediterranean territorial waters included in this study.

There are many features to consider when planning marine conservation within territorial waters. Israel has a relatively small territorial water area with a large number of marine activities (Fig. 3). In addition to the fishing and hydrocarbon operations (included as opportunity cost) we included eight additional marine activities. These include: aquaculture, desalination plants, dive sites, current protected areas, exploration safety zone (500 m buffer around hydrocarbon exploration sites), military areas (fire zones), shipping lanes and pipelines (Table 1; see File S2. for a full description of these activities and their data sources). We included these other activities by assigning their usage to specific zones (see Table 1).

### Systematic planning tools and planning scenarios

Marxan with Zones is a conservation decision-support tool that enables the user to prioritize places for different zones to achieve multiple objectives [25], [27]. This tool is an extension of Marxan [55], a globally used conservation planning tool for marine and terrestrial realms [27]. Marxan works by minimizing one variable (e.g., the opportunity cost of commercial fishing), creating a system that is separated into areas which are protected or non-protected [25]. In comparison, Marxan with Zones can minimize multiple variables (i.e. incorporating more than two opportunity cost layers) and enables the user to develop a more complex system of zones that provide varying degrees of protection and have zone specific actions, objectives and restrictions [27].

We applied Marxan [55] and Marxan with Zones [27] to compare four planning scenarios for Israel's Mediterranean territorial waters (see Table 2). For each planning scenario we aimed to meet the same biodiversity targets while minimizing the opportunity cost incurred by other marine activities, as described below. The four scenarios increase in complexity with the inclusion of human activities (threats) and economic objectives; Simple Planning, Basic Zoning, Intermediate Zoning and Complex Zoning (Table 2). We define the term “activities” as any other activity within Israel's marine waters that is not biodiversity protection as proposed in this study. For the first scenario, Simple Planning, we used Marxan (without zoning) and tested two sub-scenarios; Simple Planning A with six activities and commercial fishing opportunity cost, and Simple Planning B with seven activities and combined commercial fishing and hydrocarbon extraction opportunity cost. Our second scenario, Basic Zoning, used Marxan with Zones and included three zones and six other activities. The third scenario, Intermediate Zoning, used Marxan with Zones and included four zones and seven activities (three sub-scenarios A, B and C for protection effectiveness of the Exploration Zone; see File S2. for full explanation). In the fourth scenario, Complex Zoning, we used Marxan with Zones with four zones (for descriptions of each zone see Table 1) and ten other activities. For a detailed description of each scenario see File S2.

**Table 2:** Four planning scenarios that were examined using Marxan and Marxan with Zones for Israel's territorial Mediterranean waters.

		Planning Scenarios						
		1. Simple Planning		2. Basic Zoning	3. Intermediate Zoning			4. Complex Zoning
		A	B		A	B	C	
**Planning Tool:**	Marxan	+	+					
	Marxan with Zones			**+**	**+**	**+**	**+**	**+**
**Zones**	Conservation Zone (No-Take)			**+**	**+**	**+**	**+**	**+**
	Economic Zone (General Use)			**+**	**+**	**+**	**+**	**+**
	Benthic Protection Zone			**+**	**+**	**+**	**+**	**+**
	Exploration Zone (% of effectiveness at protecting species^3^)				+ (25%)	+ (50%)	+ (75%)	+ (50%)
**Marine activities** *(Included as opportunity cost)* ^*4*^	Commercial Fisheries: Trawlers, Purse Seiners, Gill Nets, Long liners	+	+	+	+	+	+	+
	Hydrocarbon Operations		**+**		**+**	**+**	**+**	**+**
*(Assigned to specific zones)* ^*5*^	Aquaculture	**+**	**+**	**+**	**+**	**+**	**+**	**+**
	Current Protected Areas	**+**	**+**	**+**	**+**	**+**	**+**	**+**
	Diving areas	**+**	**+**	**+**	**+**	**+**	**+**	**+**
	Military areas	**+**	**+**	**+**	**+**	**+**	**+**	**+**
	Safety platform	**+**	**+**	**+**	**+**	**+**	**+**	**+**
	Shipping lanes							**+**
	Desalination plants							**+**
	Pipelines							+
**Number of Zones**		**0**		**3**	**4**			**4**
**Total number of marine activities included in the analysis**		**6**	**7**		**7**			**10**

3 We assigned possible percentages of biodiversity protection that may be achieved by the Exploration Zone. The Conservation Zone assumes 100% protection of .biodiversity but due to the unknown impacts of hydrocarbon exploration we tested different values (25%, 50%, and 75%). See File S2. for further details.

4 The opportunity cost layers are the variables where are minimized in Marxan software: In Marxan this is treated as one minimized cost layer (summed together), and in Marxan with Zones these opportunity cost layers are separately minimized.

5 See Table 1 for the zones that each marine activity is permitted or restricted within and File S2. for a detailed explanation of each activity and their data references.

The inclusion of features and data in each scenario is represented by a plus sign (+). Planning scenarios increase (from the Simple Planning to Complex Zoning scenario) in complexity by the planning tool, zones and number of activities included. For more detailed information on each of the zones see Table 1.

### Comparing planning scenarios

Four planning scenarios (Table 2) were compared. The Simple Planning scenario (without zoning) was run using Marxan and the other three scenarios (Basic Zoning, Intermediate Zoning and Complex Zoning) used Marxan with Zones, all scenarios with 1,000 runs each. Based on the results of the 1,000 runs we calculated the average opportunity cost and number of 1 km^2^ planning units within each zone that were needed to meet our biodiversity targets. We tested the ability of planning scenarios to meet all biodiversity targets. In cases where targets were unable to be reached for a particular species, we eliminated the constraint for 5% of their protection to be met in the Conservation Zone. Thus, we re-ran our results with the same altered targets for all scenarios. We then mapped the selection frequency outputs (number of time a planning unit is selected in Marxan for a particular zone) for each planning scenario and each zone. To compare between zoning configurations and scenarios we also mapped the best solution that Marxan could find. To test the similarity between the selection frequency outputs for each scenario we used the Spearman Rank Correlation (ρ). Higher values indicate a more similar spatial pattern in selection frequencies, meaning that these plans will require similar conservation actions.

### Evaluating trade-offs

We evaluated the trade-off between meeting biodiversity targets and maximizing annual fishery revenue for each of the four fisheries in Israel's Mediterranean Sea, following methods described in Klein et al. [25]. These trade-offs can only be evaluated for scenarios using Marxan with Zones that enables multiple variables to be considered. We set fishery targets where we aimed to preserve an equal percentage of the total fishing revenue (from the fishing effort maps) for each of the four fishery gear types. These targets could only be met within zones that did not restrict that type of fishery (Table 1). Expanding this analysis, we tested the trade-off with areas that are leased and licensed for oil and gas (using the hydrocarbon opportunity cost layer described above). We therefore included a hydrocarbon target (preserving hydrocarbon industry revenue) as well as both biodiversity and fishery targets. Fishery targets were extracted from the previous trade-off analysis; the highest target where all biodiversity targets were met.

## Results

### Comparing planning scenarios for territorial waters

Here we compared our four planning scenarios (Table 2) by: a) the ability to reach biodiversity targets, b) the change in opportunity cost and c) the alteration of spatial conservation priorities.

#### a) Biodiversity targets

We found that meeting the same biodiversity targets became more difficult as our planning scenarios included more marine activities. The Simple Planning (without zoning and six activities) and Basic Zoning (three zones and six activities) scenarios met all biodiversity targets, the Intermediate Zoning scenario (four zones and seven activities) met 98% of targets and the Complex Zoning scenario (four zones and ten activities) met 96% of targets (Table 3). Our constraint (5% target in the Conservation Zone – no-take area) was unable to be met in the Intermediate and Complex Zoning scenarios for nine species (Table S4 in File S1.) that had restricted distribution ranges that overlapped with prospective hydrocarbon exploration areas. For each of these nine species we eliminated the constraint; however the overall biodiversity target for these nine species remained and was met within other zones. Targets were then able to be met for all planning scenarios.

**Table 3:** Results showing average opportunity cost for 1,000 Marxan runs for each planning scenario.

Planning Scenario	Opportunity cost (US$ million)	Percent of targets met	Percent of conservation zone in entire reserve design (no-take areas)
Simple Planning A	2.05	100	22
Simple Planning B	595,132.38	100	21
**Marxan with Zones**			
Basic Zoning	4.09	100	22
Intermediate Zoning B 50%	333,004.37	98	17
Complex Zoning	4.20	96	14
**Marxan with Zones (minus nine species for the 5% Conservation Zone target)**			
Basic Zoning	3.92	100	22
Intermediate Zoning A 25%	5.59	100	18
Intermediate Zoning B 50%	5.01	100	17
Intermediate Zoning C 75%	4.76	100	17
Complex Zoning	5.32	100	14

The constraint/target that 5% of the distribution of all features needs to be within the Conservation Zone (no-take zone) was unable to be reached for nine species. This constraint was removed for these species so targets could all be met. This table shows the opportunity cost of each planning scenario, the percentage of biodiversity targets met in the scenario and the percentage of “no-take area” surface coverage of the entire reserve system. For a description of planning scenarios see Table 2.

Targets were set according to IUCN criteria and the size of a species distribution range (as described in the methods section).

#### b) Opportunity cost

We found that more complex planning scenarios incurred greater opportunity cost (Table 3). When comparing the two Simple Planning scenarios (Simple Planning A with six users and commercial fishing opportunity cost, and Simple Planning B with seven users and combined commercial fishing and potential hydrocarbon opportunity cost) we found that a reserve network that only included the opportunity cost of fishing had a substantially lower cost (Simple Planning A = US$2.05 million) compared to a plan that included the opportunity cost of hydrocarbon operations (Simple Planning B = US$595,132.38 million). Comparing our zoning scenarios (when targets are met 100% in each scenario) we found that the most expensive zoning scenario is the Intermediate Zoning scenario A that assumes the Exploration Zone can provide a zone effectiveness measure of twenty-five percent. This opportunity cost decreased as the Exploration Zone's ability to protect biodiversity (zone effectiveness) was increased to fifty percent (Intermediate Zoning scenario B 10.5% cost decrease) and seventy-five percent (Intermediate Zoning scenario C 14.9% cost decrease); allowing targets to be met more easily within the Exploration Zone. The Intermediate Zoning scenario increased opportunity cost by 27.8% from the Basic Zoning scenario (three zones and six activities) as we introduced the opportunity cost of prospective oil and gas reserves as well as a fourth zone (Exploration Zone). The Complex Zoning scenario also increased the opportunity cost of the Basic Zoning plan by 35.7% and Intermediate Zoning B plan by 6.2%.

#### c) Conservation priorities

**Figure 4:**
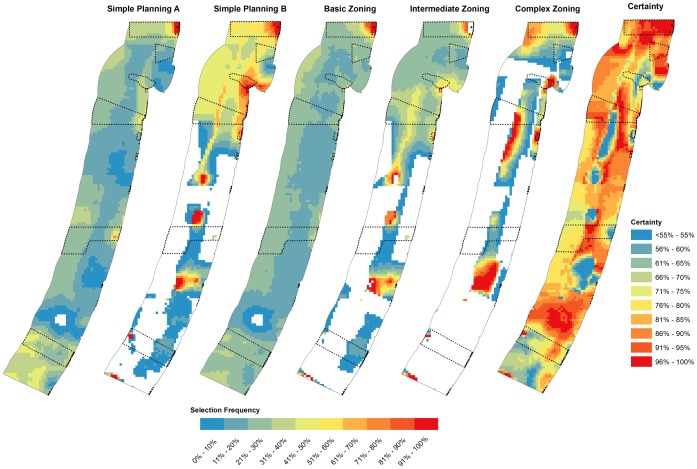
Selection frequency output maps (shows the percentage of times a planning unit was selected when run in Marxan 1000 times) from Marxan with Zones for each Zone and each zoning scenario. All scenarios meet biodiversity targets. The dashed black lines represent the proposed marine reserve system by Israel's Nature and Parks Authority [45]. The certainty map expresses the level of certainty/agreement of planning units selected (either highly selected for no-take areas or low selection) across all planning scenarios. Therefore, the higher the percentage of certainty means there is more agreement between scenarios.

**Figure 5:**
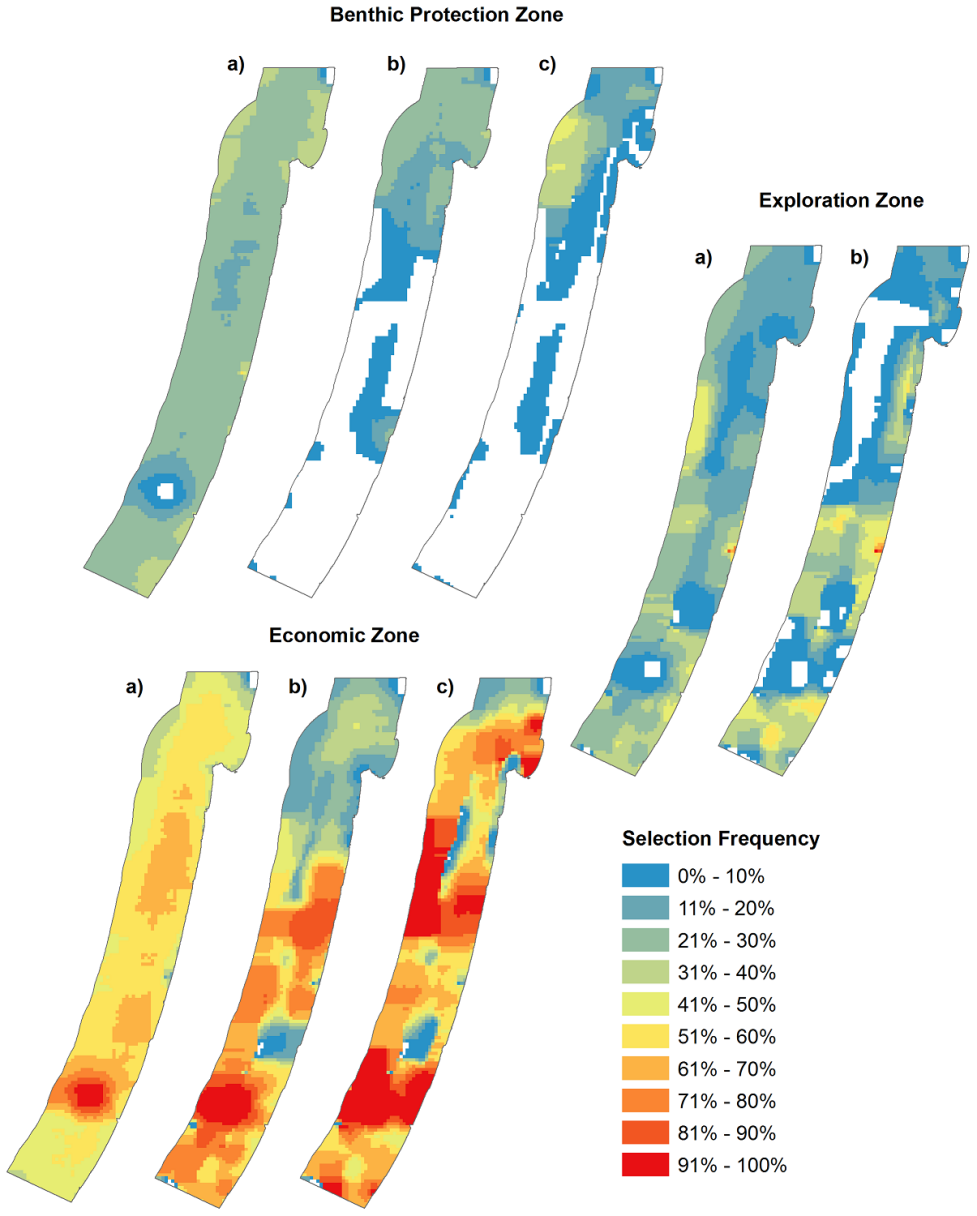
Selection frequency output maps (shows the percentage of times a planning unit was selected when run in Marxan 1000 times) from Marxan with Zones for each Zone and each zoning scenario. For the Benthic Protection Zone and Economic Zone the three scenarios are a) Basic Zoning, b) Intermediate Zoning, c) Complex Zoning. For the Exploration Zone the two scenarios are a) Intermediate Zoning and b) Complex Zoning.

Selection frequency outputs from our analysis indicated that spatial configurations are substantially altered by the inclusion of hydrocarbon opportunity cost (Fig. 4; Fig. 5). The scenarios Simple Planning A (without zoning and six activities) and Basic Zoning (three zones and six users), which did not include hydrocarbon opportunity cost had a high Spearman's rank correlation of ρ = 0.84 (p<0.001; Table 4).

**Table 4:** Spearman rank correlation (ρ) of the similarity between the selection frequency outputs of each planning scenario.

Zone	Planning Scenario	Simple Planning A	Simple Planning B	Basic Zoning	Intermediate Zoning B	Complex Zoning
Conservation Zone	Simple Planning A	-	0.69	0.84	−0.09	−0.09
	Simple Planning B	0.69	-	0.11	0.86	0.67
	Basic Zoning	0.84	0.11	-	−0.04	−0.06
	Intermediate Zoning B	−0.09	0.86	−0.04	-	0.73
	Complex Zoning	−0.09	0.67	−0.06	0.73	-
Benthic Zone	Basic Zoning	-	-	-	0.45	0.33
	Intermediate Zoning B	-	-	0.45	-	0.82
	Complex Zoning	-	-	0.33	0.82	-
Economic Zone	Basic Zoning	-	-	-	0.41	0.29
	Intermediate Zoning B	-	-	0.41	-	0.58
	Complex Zoning	-	-	0.29	0.58	-
Exploration Zone	Intermediate Zoning B	-	-	-	-	0.42
	Complex Zoning	-	-	-	0.42	-

High values (closer to 1) indicate a more similar spatial pattern in selection frequencies, meaning that these plans will require similar conservation actions. All scenarios show significant correlations (p<0.001).

**Figure 6:**
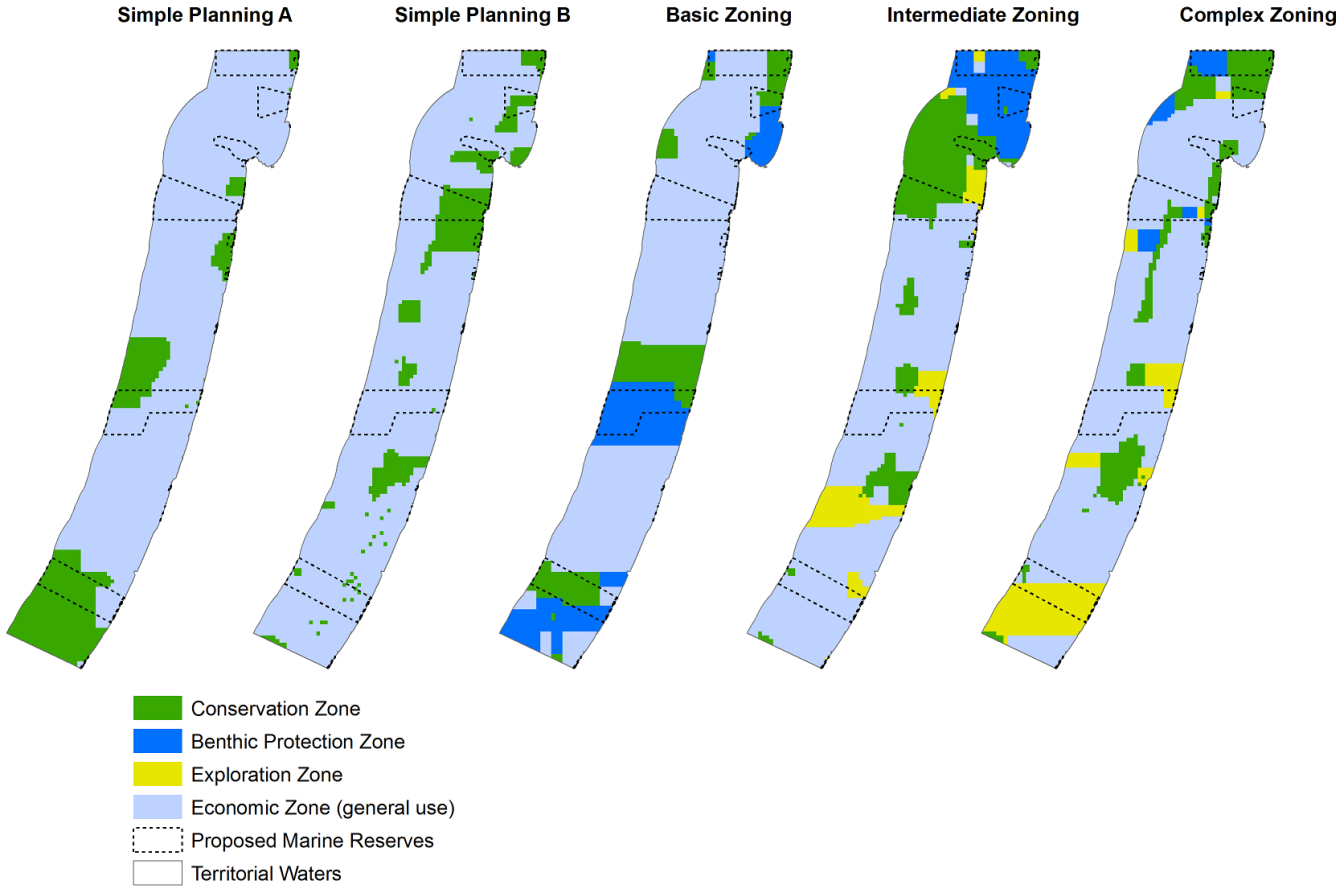
Marxan best solution outputs (the reserve configuration that best reduces opportunity cost and meets biodiversity targets from 1000 Marxan runs) for each planning scenario. The four colours designate the four types of zones (see Table 1).

We found that priority areas for no-take reserves (Conservation Zone) were mainly concentrated in the north and south of Israel's territorial waters. From the best solution outputs (Fig. 6) no-take areas moved from areas in the south to areas in the north with the inclusion of potential hydrocarbon extraction data. Similarly, the three scenarios that included hydrocarbon opportunity cost (Simple Planning B, Intermediate Zoning and Complex Zoning) had selection frequency outputs that were significantly correlated (Table 4). The most similar spatial outputs were between Simple Planning B and Intermediate Zoning B (ρ = 0.86, p<0.001; Table 4). In these three scenarios, we discovered that spatial priorities were much more distinct (higher spectrum of selection frequency; see Fig. 4) than the Simple Planning A and Basic Zoning scenarios. Areas with high selection frequency for placing no-take reserves were off the coast of Jaffa, in coastal waters between Dor and Haifa Bay and along northern border with Lebanon (Fig. 4).

Priority areas for each zone become more pronounced as planning scenarios became more complex and restricted by the inclusion of other marine activities. Conservation priorities for the Benthic Zone were most similar between the Intermediate Zoning B and Complex Zoning (ρ = 0.82, p<0.001; Table 4). In all scenarios we find that Benthic Protection Zone has higher selection frequency in the northern part of the Sea. In the best solution outputs we also notice how benthic protection becomes confined to the north with the inclusion of the Exploration Zone (Fig. 6). The Economic Zone has highest selection frequency in the south for the Basic Zoning scenario where high fishing pressure is evident. In the Intermediate Zoning B scenario the high selection frequency of this zone extends over the south and central region where hydrocarbon is included. Further expansion of this zone's high selection frequency extends to the north as shipping lanes and pipelines are included in the Complex Zoning scenario. The Exploration Zone's priority areas were dissimilar between the Intermediate and Complex Planning scenarios, (ρ = 0.42, p<0.001; Table 4). The inclusion of other marine activities affected the available area for the Exploration Zone.

### Evaluating trade-offs between conservation and economic objectives

**Figure 7:**
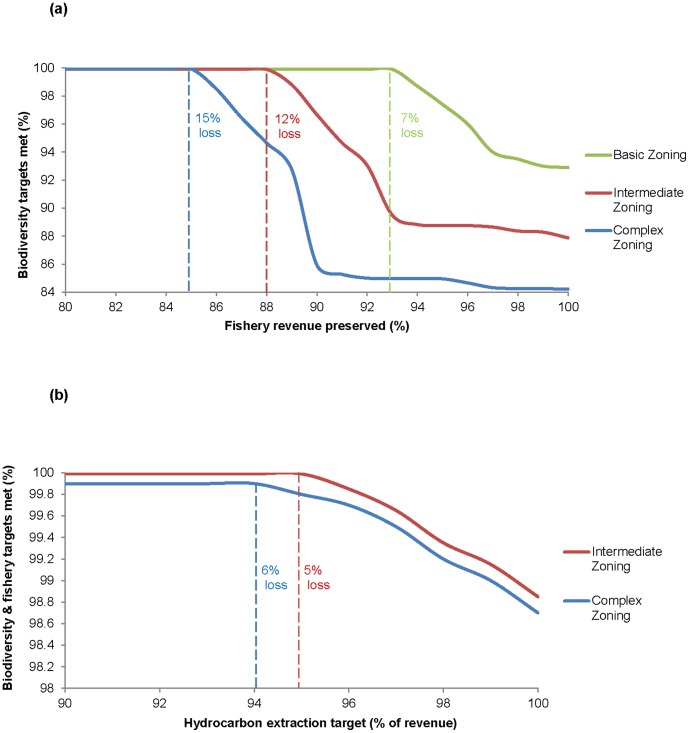
The trade-off between meeting biodiversity targets and maintaining economic objectives for each zoning scenario. (a) biodiversity targets are met when the fishery targets (percentage of annual fishery revenue) are less than 93% (7% revenue loss) in the Basic Zoning scenario (three zones and six activities), less than 88% (12% revenue loss) in the Intermediate Zoning B scenario (four zones and seven activities), and less than 85% (15% revenue loss) is the Complex Zoning scenario (four zones and ten activities), (b) biodiversity targets are met when hydrocarbon operations (leased and licensed expected revenue) are less than ≤95% (5% revenue loss) in the Intermediate Zoning scenario and less than 94% (6% revenue loss) in the Complex Zoning scenario.

In the Basic Zoning scenario (three zones and six users) all biodiversity targets were met with a loss of 7% of commercial fishing revenue (Fig. 7a). By increasing the complexity of our planning scenarios (including more marine activities) we found that our biodiversity targets could only be met by decreasing the area of fishery grounds, consequently decreasing the revenue. Hence, the resulting fishing revenue loss was 12% for the Intermediate Zoning scenario (zoning network that includes four zones) and 15% for the Complex Zoning scenario.

In comparison, by including the economic objectives of hydrocarbon operations while meeting biodiversity and fishery targets (all four fishing gear types targeted 88% (Intermediate Zoning) and 85% (Complex Zoning) of revenue; values obtained from Fig. 7a), a small revenue loss was incurred (Fig. 7b). For the Intermediate Zoning scenario 5% of hydrocarbon revenue was lost. Similarly, the Complex Zoning scenario kept biodiversity and fishery targets with revenue losses of 6%. Therefore, for a loss of ∼5% of hydrocarbon revenue, fishery and biodiversity targets could be fully met. Interestingly, we found that the drop-off rate of not meeting biodiversity and fishery targets was very minimal for the hydrocarbon industry in comparison with the rate at which biodiversity and fishery targets were traded off. Moreover, if hydrocarbon revenue was not traded-off (100% revenue was maintained), biodiversity and fishery targets could reach ∼98% (Fig. 7b). However, if fishery revenue was not traded off (100% revenue was maintained), biodiversity targets could only reach between 93–84% (Fig. 7a).

## Discussion

This study demonstrates how conservation objectives can be achieved while considering economic objectives where there are multiple marine activities. We found that the inclusion of many activities in marine conservation plans can significantly alter spatial priorities (Table 4; Fig. 4; Fig. 5). Economic goals are more compromised (in this case for the fisheries and hydrocarbon industries; Fig. 7) to achieve biodiversity targets when there are more marine activities in the planning process. Moreover, complex plans with more activities involved greater opportunity cost and did not reach biodiversity targets as easily as more simplified plans with less marine activities. Given that a complex plan is working with a more constrained problem, this result is expected [3], [56]. Despite the increased opportunity cost and lack of spatial flexibility to achieve biodiversity goals with more complex conservation plans, planning that incorporates other activities can steer us towards areas which are feasible (greater potential for implementation success), minimize conflict with other users and reduce threats to biodiversity.

Conservation planning and zoning with multiple activities is challenging. Our case study shows that decisions made by conservation planners such as the number of zones or number of marine activities included in the planning process can substantially shape the resulting zone and reserve configuration. Therefore, it is important to first identify the impact that each activity and feature has on marine biodiversity in the study system and the appropriate conservation action to take [57]. Here we follow a framework (Fig. 1) to help conservation planners address offshore activities and their potential threat in the marine realm. This framework outlines the steps needed to comprehensively zone for biodiversity protection while maintaining economic goals and can be a useful guide for countries currently striving to zone their waters [18]. One of the most important steps is testing the sensitivity of the results to user decisions (e.g., the inclusion of data, number of zones, the targets, see Step 7 Fig. 1; [58]). Other challenges that need to be accounted for when zoning include: the lack of shared information between stakeholders [59], the unknown expansion and objectives of industries [60], the unknown value of economic industries [5], and unforseen threats or disasters [18]. Given that some of these challenges can be overcome, in reality, conservation planning is largely shaped by the willingness to trade-off economic and conservation objectives. Moreover, there is no one correct solution to planning within a complex system [61].

Trade-off analysis is an important step to include in conservation planning [62], [63]. It enables us to determine how much of a commercial activity must be forgone in order to achieve biodiversity targets. It also helps to address the implementation gap (the gap between conservation planning and real-world action) inherent in many conservation plans [2]. However, Hirsch et al. [62] cautions that not every problem can be solved by compromise. For example, we assume in our study that a portion of the hydrocarbon leased and licensed areas and commercial fishing grounds are available for trade-off, whereas stakeholders may disagree and reject any compromise. In marine conservation, fishing trade-offs have been the focus of several studies [3], [25], [28]. In this study we have incorporated economic trade-offs for both the commercial fishing and hydrocarbon industries, indicating the necessary compromises that are needed to meet our biodiversity targets in each planning scenario. Specifically, we have triaged targets for nine species that were unable to be met within no-take zones (of the Intermediate Zoning and Complex Zoning scenarios) and enabled them to be met within other zones. We suggest that future work should expand this type of analysis to examine the trade-offs with other social, economic and cultural activities where appropriate.

Marine features and activities which are confined in space may be difficult, or impossible, to trade-off. In this study we introduced a range of features into marine conservation planning in addition to the more traditionally used fishing such as pipelines, shipping lanes, desalination plants and aquaculture. In comparison to the full coverage of commercial fishing practises and the wide cover of hydrocarbon exploration across the study area, other features are restricted to a specific area (Fig. 3). Such restricted features are difficult to plan around as they often cannot be traded-off. Fishing effort for example can be redispersed to other spatial areas when an area is declared a marine reserve [64], [65], whereas aquaculture farms are more difficult to relocate. We also found that linear-shaped features such as pipelines and shipping lanes influence the shape of marine zones, causing thin elongated zones (Fig. 6). Therefore, conservation planners must decide whether such features are planned around, planned with, or ignored. Performing a cost-benefit analysis of altering some of these features (e.g., rerouting shipping lanes, planning reserves over pipelines or moving planned aquaculture cages) within various planning scenarios could be a way to examine their potential flexibility within the reserve system. We suggest that future research explores the way that such features are included in conservation planning as they can have an influence on the selection of conservation priorities.

We found that the incorporation of oil and gas exploration can substantially alter spatial priorities and the opportunity cost of conservation. This is first time that offshore hydrocarbon operations are explicitly incorporated in marine conservation planning. Possible reasons for its absence in previous conservation plans are because a) the economic gains that are at stake are so large that these areas are “off-limits” to all other marine activities (e.g., Australian commonwealth zoning plan; [12]), b) uncertainty as to how to incorporate hydrocarbon information and c) the uncertain future of the industry that is dependent on new discoveries and may quickly demand large marine space (e.g., new discoveries in the Mediterranean Sea [66]). If we incorporate hydrocarbon information by assuming such areas cannot be protected, we may not be able to achieve a representative reserve network. One of the problems we encountered with including prospective hydrocarbon exploration is that sometimes biodiversity targets could not be achieved because a few species substantially overlap with hydrocarbon interests. Thus, we must carefully assess our targets and understand the compromises or actions that need to be taken in order to ensure conservation-worthy species are maintained in the face of hydrocarbon operations. We suggest that conservation plans endeavour to incorporate mining and fossil fuel data where possible to avoid costly conservation mistakes.

The ability of hydrocarbon exploration areas to provide some level of protection for biodiversity is unknown. In this study we tested different levels of protection from the “Exploration Zone” (see Intermediate Zoning Table 3; “zone effectiveness” see [67]), and found that opportunity cost is reduced if hydrocarbon areas are able to contribute to biodiversity protection. This is a novel conservation planning example that incorporates the notion of hydrocarbon areas providing some conservation benefit. The impacts of oil spills and gas leaks on marine biodiversity are severe and are well documented [68]–[70]. Likewise, there is some understanding of the impacts of offshore construction and extraction e.g., drilling impacts that are damaging to benthic structures [71]. However we have little understanding of the impacts posed by the ongoing maintenance of a drilling site that is dormant (leased or licensed without current activity). We suggest that future research focuses on better understanding the impacts that hydrocarbon operations pose on marine biodiversity and further develop ways to include hydrocarbon information into marine conservation plans.

Our study has interesting implications for Israel. We found that for Israel's territorial waters we can meet all our biodiversity targets (but not all no-take zone targets) for a loss of ∼15% of annual commercial fishery revenue and ∼5% of potential hydrocarbon revenue. A reduction of 7% of fishery revenue was needed to meet our biodiversity targets if hydrocarbon exploration is ignored. Our planning scenarios indicate that a surface area of 14–22% (Table 3) of Israel's territorial waters needs to be protected to meet biodiversity targets. The marine area reserved in Israel is currently less than 1% of the territorial waters [72], although none of these are considered no-take areas. Efforts are currently being undertaken to expand Israel's reserve network. The proposed network has been planned using species gradients and the representation of geomorphological features, without the use of Marxan or other similar tools [72]. We found that there is some overlap between the proposed marine reserves and the high priority areas found in our study (Fig. 4). The primary overlapping areas include: the proposed reserve in the north (Rosh Hanikra), the Haifa headland, the proposed reserve near Atlit and the smaller sized reserve near Dor. While different methods have been used for these two plans, some results are overlapping and we recommend that these areas that overlap should be targeted as initial reserve priorities for Israel as they are robust to the kind of process used to define priorities. However, it should be cautioned that, while overlapping priorities could be a good starting point, they will not necessarily provide a representative network that meets biodiversity targets.

Marine conservation planning often lacks good quality spatial data and must therefore rely on surrogate measures [6], [59], [73]. In this study, the surrogate fishing effort layers were generated with large involvement and input from experts. In comparison, our opportunity cost layer for hydrocarbon operations, although based on available government data, may less accurately reflect unpredictable shifts in future opportunity cost due to the fluctuating price of fossil fuels. Here we also set relatively low biodiversity targets because very minimal marine protection exists in this area, thus, these targets are potentially achievable (20% of Israel's Mediterranean Sea needs to be protected to meet our biodiversity targets (Table 3), corresponding with Israel's proposed target by the Israel Nature and Parks Authority [72]). These targets do not guarantee species persistence, but increasing these target may mean that other targets become unachievable, particularly within the Intermediate Zoning and Complex Zoning scenarios. We have included several novel features in our planning (e.g., aquaculture farms, desalination plants, shipping lanes and pipelines), yet there are other features that could be incorporated in future work, for example sand mining, offshore power plants, tourism and recreational fishing. Similarly, management and monitoring cost can also be included in future studies [6]. The aim of this study was to evaluate the impact of including multiple features and activities into marine conservation, however we do intend for these results to serve as useful baseline plans for the territorial waters of Israel. To improve the selection of conservation priorities in Israel's Mediterranean waters future work should attempt to build upon these scenarios, including additional species data for which data is currently limited, incorporate additional marine activities and create more robust cost layers with the availability of new data.

This case study can serve as an example for many other countries around the world, which are faced with the need to carefully balance economic considerations while protecting marine biodiversity. It is particularly relevant for countries surrounding the Mediterranean Sea that share common challenges and arising threats from developing offshore hydrocarbon exploration to biodiversity and ecosystems [66]. Our results suggest that planning with more complexity (e.g., multiple economic objectives, multiple threats and multiple zones) will be slightly more costly, have higher trade-offs with other marine activities and will require more input data. Despite these inefficiencies, a complex plan considers the objectives of more stakeholders (marine activities) and is more likely to result in successful implementation of conservation outcomes [2] and better compliance than a plan which ignores other activities. In the Mediterranean region with its many marine users, this is particularly important where compliance is often a major limiting factor in reserve design and implementation success [74]. We propose that countries aiming to protect marine biodiversity in their territorial waters should move from a single objective approach to one that links to the broader socioeconomic context incorporating multiple activities. A way forward may be the incorporation of lessons from marine spatial planning [11], [19] into marine conservation planning, while aiming at maintaining biodiversity goals and examining trade-offs. Explicitly quantifying trade-offs can provide an initial starting point for discussion between stakeholders [62] and ultimately enable successful conservation outcomes which other marine users are willing to comply with.

## Supporting Information

File S1 **Figures and tables (Table S1, Table S2, Tables S3, Tables S4, Figure S1, Figure S2, Figures S3).**
(DOCX)

File S2 **Supplementary material text.**
(DOCX)
