# Supplementary material for: The Crowded Sea: Incorporating Multiple Marine Activities in Conservation Plans Can Significantly Alter Spatial Priorities
Source: PLoS One. 2014 Aug 7;9(8):e104489. doi: 10.1371/journal.pone.0104489 (PMC4125186; doi:10.1371/journal.pone.0104489)
Supplement: File S1 — Figures and tables (Table S1, Table S2, Tables S3, Tables S4, Figure S1, Figure S2, Figures S3). (DOCX) [file pone.0104489.s001.docx]

**File S1. Supplementary Material**

**Table S1.** Conservation targets for each 166 conservation feature (153 fish species, 2 sea turtle species, one cetacean and 10 geomorphologic features) were set using the IUCN red listings for species; 10% target was set for all species, 15% target was set for IUCN “Vulnerable” listed species, 20% target for IUCN “Endangered” species and for “Endangered” that have <1% of the area of the territorial waters a target of 50% was set. A 5% target was set for all geomorphologic features and features with <1% of area were given a 10% target. Zones: Economic Zone (general) effectiveness = 0%; No-Take Zone effectiveness = 100% Benthic Protection zone effectiveness = Fish base values for fishes, Turtles (Donlan *et al.* 2010), Marine mammals (Coll *et al.* 2010), geomorphologic structures (we set 50% due to unknown zone effectiveness) Exploration Zone effectiveness = 3 scenarios 25%, 50% and 75%.

| **Conservation features** | **Vulnerability** | | **Benthic Protection Zone effectiveness** | **Overall Targets (IUCN)** |
| --- | --- | --- | --- | --- |
| ***Fish Species*** | | | |  |
| *Aidablennius sphynx* | Low vulnerability (16 of 100; FishBase) | | 84% | 10% |
| *Alectis alexandrinus* | Moderate to high vulnerability (50 of 100; FishBase) | | 50% | 10% |
| *Anthias anthias* | Moderate vulnerability (38 of 100; FishBase) | | 62% | 10% |
| *Apogon imberbis* | Low vulnerability (15 of 100; FishBase) | | 85% | 10% |
| *Argentina sphyraena* | Moderate vulnerability (36 of 100; FishBase) | | 64% | 10% |
| *Argyrosomus regius* | Very high vulnerability (79 of 100; FishBase) | | 21% | 10% |
| *Ariosoma balearicum* | Low to moderate vulnerability (31 of 100; FishBase) | | 69% | 10% |
| *Arnoglossus kessleri* | Low vulnerability (21 of 100; FishBase) | | 79% | 10% |
| *Arnoglossus laterna* | Moderate vulnerability (36 of 100; FishBase) | | 64% | 10% |
| *Atherina boyeri* | Moderate vulnerability (43 of 100; FishBase) | | 57% | 10% |
| *Auxis rochei* | Low to moderate vulnerability (34 of 100; FishBase). | | 66% | 10% |
| *Balistes capriscus (old name Balistes carolinensis)* | Low to moderate vulnerability (32 of 100; FishBase). | | 38% | 10% |
| *Blennius ocellaris (ocelatus)* | Low to moderate vulnerability (30 of 100; FishBase). | | 70% | 10% |
| *Boops boops* | Moderate vulnerability (41 of 100; FishBase). | | 59% | 10% |
| *Bothus podas* | Moderate to high vulnerability (51 of 100; FishBase). | | 49% | 10% |
| *Belone belone* | Moderate vulnerability (39 of 100; FishBase). | | 61% | 10% |
| *Callionymus risso* | Low vulnerability (16 of 100; FishBase) | | 84% | 10% |
| *Capros aper* | Low vulnerability (16 of 100; FishBase). | | 84% | 10% |
| *Caranx crysos* | Low to moderate vulnerability (34 of 100; FishBase). | | 66% | 10% |
| *Caranx rhonchus* | Moderate vulnerability (36 of 100; FishBase). | | 64% | 10% |
| *Carcharhinus obscurus (catgalligious)* | Vulnerable – IUCN | | 12% | 15% |
| *Cepola macrophthalma (was Cepola rubescens)* | Moderate to high vulnerability (50 of 100; FishBase) | | 50% | 10% |
| *Chelidonichthys lucernus (was lucerna and Triga lucerna)* | High vulnerability (58 of 100; FishBase) | | 42% | 10% |
| *Chelon labrosus* | Least Concern – IUCN | | 37% | 10% |
| *Chlorophthalmus agassizii* | Low to moderate vulnerability (32 of 100; FishBase) | | 68% | 10% |
| *Chromis chromis* | Moderate vulnerability (35 of 100; FishBase). | | 65% | 10% |
| *Chromogobius quadrivittatus* | Low vulnerability (20 of 100)  Least Concern – IUCN (Endemic) | | 80% | 10% |
| *Chromogobius zebratus* | Least Concern – IUCN (Endemic) Low vulnerability (15 of 100) | | 85% | 10% |
| *Citharus linguatula* | Moderate vulnerability (36 of 100; FishBase). | | 64% | 10% |
| *Clinitrachus argentatus* | Low vulnerability (10 of 100; FishBase). | | 90% | 10% |
| *Coelorhynchus coelorhynchus* | High vulnerability (62 of 100; FishBase). | | 38% | 10% |
| *Conger conger* | Very high vulnerability (86 of 100; FishBase). | | 14% | 10% |
| *Coris julis* | High vulnerability (60 of 100)  Least Concern - IUCN | | 40% | 10% |
| *Coryphoblennius galerita* | Low vulnerability (20 of 100; FishBase). | | 80% | 10% |
| *Dactylopterus volitans* | Low to moderate vulnerability (31 of 100; FishBase). | | 69% | 10% |
| *Dasyatis chrysonota (catgalligious)* | Least Concern – IUCN   High to very high vulnerability (70 of 100) | | 30% | 10% |
| *Dasyatis pastinaca (catgalligious)* | Very high vulnerability (82 of 100; FishBase). | | 18% | 10% |
| *Deltentosteus quadrimaculatus* | Low vulnerability (20 of 100; FishBase). | | 80% | 10% |
| *Dentex gibbosus* | High vulnerability (60 of 100; FishBase). | | 40% | 10% |
| *Dentex macrophthalmus* | Moderate to high vulnerability (51 of 100; FishBase). | | 49% | 10% |
| *Dentex maroccanus* | Moderate to high vulnerability (47 of 100; FishBase). | | 53% | 10% |
| *Dicentrarchus labrax* | Least Concern – IUCN; High vulnerability (57 of 100) | | 43% | 10% |
| *Diplodus annularis* | Moderate vulnerability (42 of 100; FishBase). | | 58% | 10% |
| *Diplodus cervinus* | High to very high vulnerability (69 of 100; FishBase). | | 31% | 10% |
| *Diplodus sargus* | High vulnerability (63 of 100; FishBase). | | 37% | 10% |
| *Diplodus vulgaris* | Low to moderate vulnerability (33 of 100; FishBase). | | 67% | 10% |
| *Echelus myrus* | Moderate to high vulnerability (49 of 100; FishBase). | | 51% | 10% |
| *Echeneis naucrates* | Moderate to high vulnerability (54 of 100; FishBase). | | 46% | 10% |
| *Echiodon dentatus* | Least Concern – IUCN; Low vulnerability (10 of 100) | | 90% | 10% |
| *Enchelycore anatina* | High vulnerability (59 of 100; FishBase). | | 41% | 10% |
| *Engraulis encrasicolus* | Low vulnerability (14 of 100; FishBase). | | 86% | 10% |
| *Epinephelus aeneus* | Near threatened – IUCN; Moderate to high vulnerability (52 of 100) | | 48% | 10% |
| *Epinephelus costae* | High to very high vulnerability (66 of 100; FishBase). | | 34% | 10% |
| *Epinephelus marginatus* | Endangered – IUCN; High to very high vulnerability (72 of 100) | | 28% | 20% |
| *Euthynnus alletteratus* | Least Concern – IUCN; High vulnerability (57 of 100) | | 43% | 10% |
| *Gnathophis mystax* | Moderate vulnerability (44 of 100; FishBase). | | 56% | 10% |
| *Gobius bucchichi* | Least Concern – IUCN  Low vulnerability (15 of 100) | | 85% | 10% |
| *Gobius cobitis* | Moderate vulnerability (39 of 100; FishBase). | | 61% | 10% |
| *Gobius cruentatus* | Low to moderate vulnerability (31 of 100; FishBase). | | 69 | 10% |
| *Gobius fallax* | Least Concern – IUCN  Low vulnerability (22 of 100) | | 78% | 10% |
| *Gobius niger* | Moderate vulnerability (38 of 100; FishBase). | | 62% | 10% |
| *Gobius pagenllus* | Low vulnerability (19 of 100; FishBase). | | 81% | 10% |
| *Gouania willdenowi* | Low to moderate vulnerability (27 of 100; FishBase). | | 73% | 10% |
| *Gymnothorax unicolor* | Moderate to high vulnerability (50 of 100; FishBase). | | 50% | 10% |
| *Helicolenus dactylopterus* | Moderate to high vulnerability (52 of 100; FishBase). | | 48% | 10% |
| *Hippocampus guttulatus* | Low vulnerability (24 of 100; FishBase). | | 76% | 10% |
| *Hypleurochilus bananensis* | Least concern – IUCN *Endemic   Low to moderate vulnerability (26 of 100) | | 74% | 10% |
| *Lepadogaster candollii* | Moderate vulnerability (38 of 100; FishBase). | | 62% | 10% |
| *Lepadogaster lepadogaster* | Least concern – IUCN *Endemic | | 64%  Moderate vulnerability (36 of 100) | 10% |
| *Lepidopus caudatus* | Moderate to high vulnerability (54 of 100; FishBase). | | 66% | 10% |
| *Lepidotrigla cavillone* | Low vulnerability (25 of 100; FishBase). | | 75% | 10% |
| *Lesuerigobius suerii* | Low vulnerability (12 of 100; FishBase). | | 88% | 10% |
| *Lipophrys canevae* | Low vulnerability (15 of 100; FishBase). | | 85% | 10% |
| *Lipophrys pavo changed to Salaria pavo* | Low to moderate vulnerability (25 of 100; FishBase). | | 75% | 10% |
| *Lipophrys trigloides* | Low vulnerability (24 of 100; FishBase). | | 76% | 10% |
| *Lithognathus mormyrus* | Moderate vulnerability (40 of 100; FishBase). | | 60% | 10% |
| *Liza aurata* | Least concern – IUCN  Moderate to high vulnerability (50 of 100) | | 50% | 10% |
| *Liza ramada* | Least concern – IUCN  Moderate vulnerability (37 of 100) | | 63% | 10% |
| *Macrorhamphosus scolopax* | Least concern – IUCN Low to moderate vulnerability (27 of 100) | | 73% | 10% |
| *Merluccius merluccius* | High vulnerability (65 of 100; FishBase). | | 35% | 10% |
| *Microchirus ocellatus* | Low vulnerability (25 of 100; FishBase). | | 75% | 10% |
| *Mugil cephalus* | Least concern – IUCN  Moderate vulnerability (42 of 100) | | 58% | 10% |
| *Mullus barbatus* | Moderate vulnerability (36 of 100; FishBase). | | 64% | 10% |
| *Mullus surmuletus* | Moderate vulnerability (37 of 100; FishBase). | | 63% | 10% |
| *Muraena helena* | High to very high vulnerability (74 of 100; FishBase). | | 26% | 10% |
| *Mycetroperca rubra* | Least concern – IUCN Very high vulnerability (81 of 100) | | 19% | 10% |
| *Oblada melanura* | Low to moderate vulnerability (34 of 100; FishBase). | | 66% | 10% |
| *Oedalechilus labeo* | Moderate vulnerability (40 of 100; FishBase). | | 60% | 10% |
| *Ophiodon barbatum* | Low to moderate vulnerability (32 of 100; FishBase). | | 68% | 10% |
| *Pagellus acarne* | Moderate vulnerability (43 of 100; FishBase). | | 57% | 10% |
| *Pagellus bogaraveo* | High to very high vulnerability (70 of 100; FishBase). | | 30% | 10% |
| *Pagellus erythrinus* | Moderate to high vulnerability (54 of 100; FishBase). | | 46% | 10% |
| *Pagrus coeruleostictus* | Moderate to high vulnerability (47 of 100; FishBase). | | 53% | 10% |
| *Pagrus pagrus* | Endangered - IUCN  High to very high vulnerability (66 of 100) | | 34% | 20% |
| *Parablennius gattorugine* | Least concern – IUCN  Low vulnerability (21 of 100) | | 79% | 10% |
| *Parablennius incognitus* | Low vulnerability (14 of 100; FishBase). | | 86% | 10% |
| *Parablennius rouxi* | Least concern – IUCN Low vulnerability (16 of 100) | | 84% | 10% |
| *Parablennius saguinolentus* | Low to moderate vulnerability (30 of 100; FishBase). | | 70% | 10% |
| *Parablennius tentacularis* | Low to moderate vulnerability (26 of 100; FishBase). | | 74% | 10% |
| *Parablennius zvonimiri* | Low vulnerability (14 of 100; ; FishBase) *Endemic | | 86% | 10% |
| *Phycis blennoides* | High vulnerability (58 of 100; FishBase). | | 42% | 10% |
| *Pomadasys incisus* | Least concern – IUCN Moderate vulnerability (40 of 100) | | 60% | 10% |
| *Raja clavata (catgalligious)* | Near threatened – IUCN Very high vulnerability (76 of 100) | | 24% | 10% |
| *Raja miraletus (catgalligious)* | Least concern – IUCN Moderate to high vulnerability (51 of 100) | | 49% | 10% |
| *Raja montagui (catgalligious)* | Least concern – IUCN High vulnerability (59 of 100) | | 41% | 10% |
| *Rhinobatos rhinobatos (catgalligious)* | Endangered – IUCN High to very high vulnerability (68 of 100) | | 32% | 20% |
| *Sardina pilchardus* | Moderate vulnerability (36 of 100; FishBase). | | 64% | 10% |
| *Sardinella aurita* | Moderate vulnerability (36 of 100; FishBase). | | 64% | 10% |
| *Sardinella maderensis* | Low to moderate vulnerability (33 of 100; FishBase). | | 67% | 10% |
| *Sargocentron rubrum* | Low vulnerability (24 of 100; FishBase). | | 76% | 10% |
| *Sarpa salpa* | Moderate vulnerability (41 of 100; FishBase). | | 59% | 10% |
| *Scartella cristata* | Low vulnerability (23 of 100; FishBase). | | 77% | 10% |
| *Sciaena umbra* | High vulnerability (64 of 100; FishBase). | | 36% | 10% |
| *Scomber japonicus* | Least concern – IUCN Moderate to high vulnerability (46 of 100) | | 54% | 10% |
| *Scorpaena elongata* | High to very high vulnerability (67 of 100; FishBase). | | 23% | 10% |
| *Scorpaena maderensis* | Moderate vulnerability (36 of 100; FishBase). | | 64% | 10% |
| *Scorpaena notata* | Moderate vulnerability (42 of 100; FishBase). | | 58% | 10% |
| *Scorpaena porcus* | Moderate to high vulnerability (49 of 100; FishBase). | | 51% | 10% |
| *Scorpaena scrofa* | High to very high vulnerability (68 of 100; FishBase). | | 32% | 10% |
| *Seriola dumerili* | Moderate to high vulnerability (54 of 100; FishBase). | | 46% | 10% |
| *Serranus cabrilla* | Moderate vulnerability (36 of 100; FishBase). | | 64% | 10% |
| *Serranus hepatus* | Low to moderate vulnerability (31 of 100; FishBase). | | 69% | 10% |
| *Serranus scriba* | Moderate vulnerability (38 of 100; FishBase). | | 63% | 10% |
| *Solea solea* | Low to moderate vulnerability (35 of 100; FishBase). | | 65% | 10% |
| *Sparisoma cretense* | Least concern – IUCN  Moderate vulnerability (36 of 100) | | 64% | 10% |
| *Sparus aurata* | Low to moderate vulnerability (35 of 100; FishBase). | | 65% | 10% |
| *Sphoeroides pachygaster* | Vulnerable – IUCN  Moderate to high vulnerability (47 of 100) | | 53% | 15% |
| *Sphyraena sphyraena* | Moderate to high vulnerability (49 of 100; FishBase). | | 51% | 10% |
| *Spicara maena* | Low to moderate vulnerability (33 of 100; FishBase). | | 67% | 10% |
| *Spicara smaris* | Moderate vulnerability (39 of 100; FishBase). | | 61% | 10% |
| *Symphodus mediterraneus* | Least concern – IUCN Low vulnerability (23 of 100) | | 77% | 10% |
| *Symphodus ocellatus* | Least concern- IUCN *Endemic  Low vulnerability (14 of 100) | | 86% | 10% |
| *Symphodus roissali* | Least concern – IUCN  Low to moderate vulnerability (31 of 100) | | 69% | 10% |
| *Symphodus tinca* | Least concern – IUCN Moderate vulnerability (37 of 100) | | 63% | 10% |
| *Synodus saurus* | Least concern – IUCN Low to moderate vulnerability (31 of 100) | | 69% | 10% |
| *Thalassoma pavo* | Least concern – IUCN Moderate vulnerability (40 of 100) | | 60% | 10% |
| *Torpedo marmorata (catgalligious)* | High to very high vulnerability (69 of 100; FishBase). | | 31% | 10% |
| *Torpedo torpedo (catgalligious)* | High to very high vulnerability (65 of 100; FishBase). | | 35% | 10% |
| *Trachinotus ovatus (catgalligious)* | Moderate vulnerability (38 of 100; FishBase). | | 62% | 10% |
| *Trachinus araneus (catgalligious)* | Moderate vulnerability (42 of 100; FishBase). | | 58% | 10% |
| *Trachinus draco (catgalligious)* | Moderate vulnerability (42 of 100; FishBase). | | 58% | 10% |
| *Trachurus mediterraneus* | Moderate to high vulnerability (46 of 100; FishBase). | | 54% | 10% |
| *Trachurus trachurus* | High vulnerability (56 of 100; FishBase). | | 44% | 10% |
| *Trichiurus lepturus* | High vulnerability (57 of 100; FishBase). | | 43% | 10% |
| *Trigloporus lastoviza* | Low to moderate vulnerability (32 of 100; FishBase). | | 68% | 10% |
| *Tripterygion delaisi* | Low vulnerability (14 of 100; FishBase). | | 86% | 10% |
| *Tripterygion melanurus* | Least concern – IUCN *Endemic  Low vulnerability (10 of 100) | | 90% | 10% |
| *Tripterygion tripteronotus* | Least concern – IUCN *Endemic  Low vulnerability (13 of 100) | | 87% | 10% |
| *Umbrina cirrosa* | Moderate vulnerability (40 of 100; FishBase). | | 60% | 10% |
| *Uranoscopus scaber* | Moderate vulnerability (44 of 100; FishBase). | | 56% | 10% |
| *Xyrichthys novacula* | Least concern – IUCN Moderate vulnerability (36 of 100) | | 74% | 10% |
| *Zebrus zebrus* | Least concern – IUCN *Endemic  Low vulnerability (13 of 100) | | 87% | 10% |
| Zeus faber | Moderate vulnerability (41 of 100; FishBase). | | 59% | 10% |
| **Sea turtle species** | | | |  |
| *Caretta caretta* | Endangered – IUCN  74% (bycatch fishing threat score from expert based survey in Donlan *et al.* 2010) | | 26% | 50% |
| *Chelonia mydas* | Endangered – IUCN  71% (bycatch fishing threat score from expert based survey in Donlan *et al.* 2010) | | 29% | 50% |
| **Cetaceans** | | | |  |
| *Tursiops truncates (Mediterranean sea sub-population* | | Vulnerable – IUCN  60% (threat analysis by Coll *et al.* 2010) | 60% | 15% |
| **Geomorphological features** | | | |  |
| Shallow rocks (25.31km^2^) | >1% of territorial waters | | 50% | 10% |
| Kukar ridges (245.14 km^2^) |  | | 50% | 5% |
| Kukar bustan (11.12 km^2^) | >1% of territorial waters | | 50% | 10% |
| Deep kukar ridges (188.64 km^2^) |  | | 50% | 5 % |
| Continental shelf silt (233.99 km^2^) |  | | 50% | 5 % |
| Continental shelf sand (2,040.98 km^2^) |  | | 50% | 5 % |
| Continental ridges (35.97 km^2^) | >1% of territorial waters | | 50% | 10% |
| Big canyons (31.80 km^2^) | >1% of territorial waters | | 50% | 10 % |
| Continental slope and canyons (585.23 km^2^) |  | | 50% | 5% |
| Deep Sea (561.07 km^2^) |  | | 50% | 5% |

**Table S2.** A list of 159 native fish species (cartilaginous fishes and bony fishes) in Israel’s territorial waters complied from eight publications with ranges and locations checked against the Hebrew University Collection. Six species were removed from this list and are marked by a * (see Appendix A2 for further information), therefore this study used a total of 153 species.

| **Native Fish species** | **Golani et al. 2007**  Site 1 | **Golani et al. 2007**  Site 2 | **Golani et al. 2007**  Site 3 | **Edelist 2010** | **Diamant 1986** | **Goren & Galil 2001** | **Yarit 2012** | **Spanier**  **2006** | **Lipsky South** | **Lipsky Center** | **Lipsky North** | **Stern 2010** | **Range as documented in the Hebrew University**  **Collection** |
| --- | --- | --- | --- | --- | --- | --- | --- | --- | --- | --- | --- | --- | --- |
| Aidablennius sphynx | X | X | X |  | X | X |  |  |  |  |  |  | RoshHaNikra - Palmachim |
| Alectis alexandrinus |  |  |  | X |  |  | X |  |  |  |  | X | Haifa - Ashqelon |
| Anthias anthias | X | X | X |  | X |  |  |  |  |  |  |  | Haifa - Yaffo |
| Apogon imberbis | X | X | X | X | X |  | X | X | X | X | X |  | RoshHaNikra - Hertziliya |
| Argentina sphyraena |  |  |  | X |  |  |  |  |  |  |  |  | Haifa-Hadera |
| Argyrosomus regius |  |  |  |  |  |  |  |  |  |  | X | X | Haifa-Yafo |
| Ariosoma balearicum |  |  |  | X |  |  | X |  |  |  |  | X | Haifa - Yaffo |
| Arnoglossus kessleri |  |  |  |  |  |  | X |  |  |  |  | X | Haifa, Yaffo |
| Arnoglossus laterna |  |  |  |  |  |  | X |  |  |  |  | X | Haifa - Ashdod |
| Atherina boyeri | X | X | X |  |  | X | X |  |  |  |  | X | Shikmona - Gaza |
| Auxis rochei |  |  |  |  |  |  |  |  |  |  |  | X | Haifa |
| Balistes capriscus (old name Balistes carolinensis) |  |  |  | X |  |  | X | X | X | X |  | X | Akko - Ashdod |
| Blennius ocellaris (ocelatus) |  |  |  | X |  |  | X |  |  |  |  |  | Haifa - Ashkelon |
| Boops boops | X | X | X | X | X | X | X | X |  |  |  | X | Haifa bay - Gaza |
| Bothus podas | X | X | X |  | X |  | X |  |  |  |  | X | Akko - Gaza |
| Belone belone |  |  |  |  |  |  |  |  |  |  |  | X | Gaza - Haifa bay |
| Callionymus risso |  |  |  |  | X |  | X |  |  |  |  |  | Yaffo |
| Capros aper |  |  |  | X |  |  |  |  |  |  |  |  | Haifa - Gaza |
| Caranx crysos | X | X | X | X | X | X | X | X |  |  |  | X | Akko - Gaza |
| Caranx rhonchus |  |  |  |  |  |  |  |  |  |  |  | X | Haifa - Gazza |
| Carcharhinus obscurus (catgalligious) |  |  |  | X |  |  |  |  |  |  |  |  | Yaffo - Ashdod |
| Cepola macrophthalma (was Cepola rubescens) |  |  |  |  |  |  | X |  |  |  |  |  | Haifa Bya |
| Chelidonichthys lucernus (was lucerna and Triga lucerna) |  |  |  |  |  |  | X |  |  |  |  | X | Haifa - Ashkelon |
| Chelon labrosus | X | X | X |  | X |  |  |  |  |  |  |  | RoshHaNikra - Tel Aviv |
| Chlorophthalmus agassizii |  |  |  | X |  |  | X |  |  |  |  |  | Haifa - Ashdod |
| Chromis chromis | X | X | X |  |  |  |  | X | X | X | X |  | Akko - Sdot Yam |
| Chromogobius quadrivittatus | X | X | X |  | X |  |  |  |  |  |  |  | RoshHaNikra - Mikhmoret |
| Chromogobius zebratus | X | X | X |  |  |  |  |  |  |  | X |  | RoshHaNikra - Neve Yam |
| Citharus linguatula |  |  |  | X |  |  | X |  |  |  |  | X | Haifa - Ashdod |
| Clinitrachus argentatus | X | X | X |  | X | X |  |  |  |  |  |  | Shiqmona - Mikhmoret |
| Coelorhynchus coelorhynchus |  |  |  | X |  |  |  |  |  |  |  |  | Haifa - Ashdod |
| Conger conger |  |  |  | X |  |  | X |  |  |  |  | X | Haifa - Ashdod |
| Coris julis | X | X | X |  | X | X |  | X | X |  |  |  | Full Coastline |
| Coryphoblennius galerita | X | X | X |  | X | X |  |  |  |  |  |  | Shiqmona - Michmoret |
| Dactylopterus volitans |  |  |  |  |  |  | X | X |  |  |  | X | Akko - Yaffo |
| Dasyatis chrysonota (catgalligious) |  |  |  |  |  |  | X |  |  |  |  |  |  |
| Dasyatis pastinaca (catgalligious) |  |  |  | X |  |  | X | X | X | 0 | 0 | X | Haifa - Nakdiman |
| Deltentosteus quadrimaculatus |  |  |  |  |  |  | X |  |  |  |  | X | Ashkelon -Hadera |
| Dentex gibbosus |  |  |  |  |  |  | X |  |  |  |  | X | Haifa - Jaffo |
| Dentex macrophthalmus |  |  |  | X |  |  |  | X |  |  |  |  | Akko - Yaffo |
| Dentex maroccanus |  |  |  |  |  |  | X |  |  |  |  |  | Haifa - Ashkelon |
| Dicentrarchus labrax |  |  |  |  |  | X |  |  |  |  |  |  | Haifa - Zikim |
| Diplodus annularis |  |  |  | X |  |  | X |  |  |  |  | X | Haifa - Jaffo |
| Diplodus cervinus | X | X | X | X | X |  | X |  | X | X |  | X | Akko - Yaffo |
| Diplodus sargus | X | X | X | X | X | X | X | X | X | X | X | X | Full Coastline |
| Diplodus vulgaris | X | X | X | X | X |  | X | X | X | X | X | X | Akko - Palmachim |
| Echelus myrus |  |  |  | X |  |  | X |  |  |  |  | X | Nahariya - Ashdod |
| Echeneis naucrates |  |  |  | X |  |  | X |  |  |  |  | X | Akko - Gaza |
| Echiodon dentatus |  |  |  |  |  |  | X |  |  |  |  |  | Hadera - Ashdod |
| Enchelycore anatina |  |  |  |  |  |  |  |  |  | X |  |  | Yaffo |
| Engraulis encrasicolus |  |  |  | X |  |  | X |  | X |  |  | X | Haifa - Gaza |
| Epinephelus aeneus | X | X | X | X | X | X | X |  | X |  |  | X | Full Coastline |
| Epinephelus costae | X | X | X |  |  |  |  |  | X |  |  |  | Akko - Gaza |
| Epinephelus marginatus | X | X | X |  | X | X |  |  | X | X |  |  | Full Coastline |
| Euthynnus alletteratus |  |  |  |  |  |  |  | X |  |  |  | X | Akko - Yaffo |
| Eutrigla gurnardus* |  |  |  |  |  |  |  |  |  |  |  | X |  |
| Gnathophis mystax |  |  |  |  |  |  | X |  |  |  |  |  | Haifa - Ashdod |
| Gobius bucchichi | X | X | X |  | X | X |  |  |  | X |  |  | RoshHaNikra - Mikhmoret |
| Gobius cobitis | X | X | X |  | X | X |  |  |  |  |  |  | Full Coastline |
| Gobius cruentatus | X | X | X |  | X |  |  |  |  | X |  |  | Haifa bay - Ceasarea |
| Gobius fallax |  |  |  |  |  |  |  |  | X |  | X |  |  |
| Gobius niger | X | X | X |  | X |  | X |  |  |  |  | X | Akko - Gaza |
| Gobius pagenllus | X | X | X |  | X | X |  |  |  |  | X |  | Full Coastline |
| Gouania willdenowi |  |  | X |  |  |  |  |  |  |  |  |  | RoshHaNikra |
| Gymnothorax unicolor | X | X | X |  | X |  |  |  |  |  |  |  | Haifa - Netanya |
| Helicolenus dactylopterus |  |  |  | X |  |  |  |  |  |  |  |  | Hertziliya - Ashdod |
| Hippocampus guttulatus |  |  |  |  |  |  | X |  |  |  |  |  | Haifa - Yaffo |
| Hypleurochilus bananensis | X | X | X |  |  |  |  |  |  |  |  |  | Akko - Michmoret |
| Lepadogaster candollii |  |  | X |  |  |  |  |  |  |  |  |  | RoshHaNikra |
| Lepadogaster lepadogaster |  |  | X |  |  |  |  |  |  |  |  |  | RoshHaNikra |
| Lepidopus caudatus |  |  |  | X |  |  |  |  |  |  |  |  | Nahariya - Ashdod |
| Lepidotrigla cavillone |  |  |  | X |  |  | X |  |  |  |  | X | Haifa bay - Ashkelon |
| Lesuerigobius suerii |  |  |  | X |  |  | X |  |  |  |  |  | Haifa - Yaffo |
| Lipophrys canevae | X | X | X |  | X | X |  |  |  |  |  |  | RoshHaNikra - Caesarea |
| Lipophrys pavo | X | X | X |  | X |  |  |  |  |  |  |  | Nahariya - Ashkelon |
| Lipophrys trigloides | X | X | X |  | X | X |  |  |  |  |  |  | RoshHaNikra - Bay Yam |
| Lithognathus mormyrus | X | X | X | X | X | X | X | X |  |  |  | X | Full Coastline |
| Liza aurata | X | X | X |  |  |  | X |  |  |  |  |  | Akko - Ashkelon |
| Liza ramada | X | X | X |  |  |  |  |  |  |  |  |  | Kishon - Gaza |
| Macrorhamphosus scolopax |  |  |  | X |  |  | X |  |  |  |  |  | Haifa - Gaza |
| Merluccius merluccius |  |  |  | X |  |  | X |  |  |  |  | X | Haifa - Ashkelon |
| Microchirus ocellatus |  |  |  | X |  |  | X |  |  |  |  |  | Haifa - Akhziv |
| Microlipophrys nigriceps (none in Golani records) * |  |  |  |  | X |  |  |  |  |  |  |  |  |
| Mugil cephalus | x | X | X |  | X |  |  |  |  |  |  |  | Akko - Hadera |
| Mullus barbatus |  |  |  | X |  |  | X |  |  |  |  | X | Akko - Ashdod |
| Mullus surmuletus | X | X | X | X | X | X | X | X |  |  | X | X | Haifa - Palmachim |
| Muraena helena | X | X | X |  |  |  |  | X |  |  |  |  | RoshHaNikra - Netanya |
| Mycetroperca rubra | X | X | X |  |  |  |  |  | X | X | X |  | RoshHaNikra - Tel Aviv |
| Oblada melanura | X | X | X | X | X |  |  | X | X |  | X |  | Full Coastline |
| Oedalechilus labeo | X | X | X |  | X | X |  |  |  |  |  |  | RoshHaNikra - Mikhmoret |
| Ophiodon barbatum |  |  |  | X |  |  | X |  |  |  |  |  | Haiffa - Yaffo |
| Pagellus acarne |  |  |  | X |  |  | X | X |  |  |  | X | Haifa - Tel Aviv |
| Pagellus bogaraveo |  |  |  | X |  |  |  |  |  |  |  |  | Hadera - Ashdod |
| Pagellus erythrinus |  |  |  | X |  |  | X | X |  |  |  | X | Haifa bay - Ashdod |
| Pagrus coeruleostictus |  |  |  | X |  |  | X | X |  | X |  | X | Akko - Yaffo |
| Pagrus pagrus |  |  |  |  |  |  | X | X |  |  |  |  | Haifa - Kishon |
| Parablennius gattorugine | X | X | X |  |  | X |  |  |  |  |  |  | Shikmona - Michmoret |
| Parablennius incognitus | X | X | X |  | X | X |  |  |  |  |  |  | Akko - Michmoret |
| Parablennius rouxi |  |  |  |  |  |  |  |  | X | X |  |  |  |
| Parablennius saguinolentus | X | X | X |  | X | X |  |  |  |  |  |  | RoshHaNikra - Palmachim |
| Parablennius tentacularis |  |  |  |  |  |  |  |  |  | X |  |  | Rosh Hanikra |
| Parablennius zvonimiri | X | X | X |  | X | X |  |  | X | X |  |  | RoshHaNikra - Mikhmoret |
| Phycis blennoides |  |  |  |  |  |  | x |  |  |  |  |  | Haifa bay - Ashdod |
| Pomadasys incisus | X | X | X | X | X |  | X |  |  |  |  | X | Haifa bay - Gaza |
| Raja clavata (catgalligious) |  |  |  | X |  |  | X |  | X |  |  |  | Hadera - Ashdod |
| Raja miraletus (catgalligious) |  |  |  | X |  |  | X |  |  |  |  | X | Haifa - Palmachim |
| Raja montagui (catgalligious) |  |  |  |  |  |  |  |  |  |  |  | X |  |
| Remora remora * |  |  |  |  |  |  |  | X |  |  |  |  | Yaffo |
| Rhinobatos rhinobatos (catgalligious) |  |  |  | X |  |  | X |  |  |  |  | X | Haifa - Tel Aviv |
| Rhinobatus cemiculus (catgalligious) * |  |  |  |  |  |  |  |  |  |  |  | X | Haifa - Ashkelon |
| Sardina pilchardus |  |  |  | X |  |  | X |  |  |  |  | X | Akko - Ashdod |
| Sardinella aurita |  |  |  | X |  |  | X | X |  |  |  | X | Akko - Askelon |
| Sardinella maderensis |  |  |  |  |  |  |  |  |  |  |  | X | Akko - Yaffo |
| Sargocentron rubrum | X | X | X | X |  |  | X | X | X | X | X | X | Full Coastline |
| Sarpa salpa | X | X | X |  | X |  |  |  |  |  |  | X | Haifa - Gaza |
| Scartella cristata | X | X | X |  | X | X |  |  |  |  |  |  | RoshHaNikra - Ashqelon |
| Sciaena umbra | X | X | X |  | X |  |  | X | X |  |  |  | Haifa - Gaza |
| Scomber japonicus |  |  |  | X |  |  | X |  |  |  |  | X | Full Coastline |
| Scomber scombrus * |  |  |  |  |  |  |  |  |  |  |  | X |  |
| Scorpaena elongata |  |  |  | X |  |  | X |  |  |  |  |  | Akko - Netanya |
| Scorpaena maderensis | X | X | X |  | X | X | X |  | X | X | X |  | Shiqmona - Ashdod |
| Scorpaena notata |  |  |  | X |  |  | X |  |  |  |  |  | Akko - Yaffo |
| Scorpaena porcus | X | X | X |  | X | X |  | X |  |  |  |  | Full Coastline |
| Scorpaena scrofa |  |  |  |  |  | X |  | X |  |  |  |  | Akko - Ashkelon |
| Seriola dumerili | X | X | X | X | X |  | X |  | X |  |  | X | Akko - Yaffo |
| Serranus cabrilla |  |  |  | X |  |  | X | X |  | X | X |  | Akko - Ashkelon |
| Serranus hepatus |  |  |  | X |  |  | X |  |  |  |  | X | Haifa bay - Ashdod |
| Serranus scriba |  |  |  |  |  | X |  | X |  |  | X | X | Akko - Gaza |
| Solea solea | X | X | X |  |  |  | X |  |  |  |  | X | Haifa - Gaza |
| Sparisoma cretense | X | X | X |  | X |  |  | X | X | X |  |  | Akko - Asheklon |
| Sparus aurata |  |  |  | X |  |  | X | X |  |  |  | X | Haifa - Gaza |
| Sprattus sprattus * |  |  |  |  |  |  |  |  |  |  |  | X |  |
| Sphoeroides pachygaster |  |  |  | X |  |  |  |  |  |  |  |  | Haifa - Ashdod |
| Sphyraena sphyraena |  |  |  | X |  |  | X | X |  |  |  | X | Haifa - Gaza |
| Spicara maena |  |  |  | X |  |  | X | X |  |  |  | X | Haifa - Ashdod |
| Spicara smaris |  |  |  | X |  |  | X | X |  |  |  | X | Haifa - Ashdod |
| Symphodus mediterraneus |  |  |  |  |  |  |  | X |  |  |  |  | Rosh - Haifa bay |
| Symphodus ocellatus | X | X | X |  | X |  |  |  |  |  |  |  | Haifa - Gaza |
| Symphodus roissali | X | X | X |  | X | X |  |  |  |  |  |  | Akko - Gaza |
| Symphodus tinca | X | X | X |  |  |  |  | X |  |  |  |  | Akko - Gaza |
| Synodus saurus |  |  |  | X |  |  | X | X |  |  | X | X | Haifa bay - Ashkelon |
| Thalassoma pavo | X | X | X |  | X | X |  | X | X | X | X |  | Akko - Gaza |
| Torpedo marmorata (catgalligious) |  |  |  |  |  |  | X |  |  |  |  | X | Akko - Ashdod |
| Torpedo torpedo (catgalligious) |  |  |  | X |  |  | X |  |  |  |  | X | Akko - Gaza |
| Trachinotus ovatus (catgalligious) | X | X | X |  |  |  |  |  |  |  |  |  | Akko - Gaza |
| Trachinus araneus (catgalligious) |  |  |  |  |  |  | X |  |  |  |  | X | Akko - Yaffo |
| Trachinus draco (catgalligious) | X | X | X |  | X |  | X |  |  |  |  | X | Akko - Gaza |
| Trachurus mediterraneus |  |  |  | X |  |  | X |  |  |  |  | X | Akko - Yaffo |
| Trachurus trachurus |  |  |  | X |  |  | X | X |  |  |  | X | Hadera - Yaffo |
| Trichiurus lepturus |  |  |  | X |  |  | X |  |  |  |  |  | Akko - Yaffo |
| Trigloporus lastoviza |  |  |  | X | X |  | X |  |  |  |  |  | Haifa - Ashkelon |
| Tripterygion delaisi | X | X | X |  |  | X |  |  |  |  |  |  | Shiqmona - Mikhmoret |
| Tripterygion melanurus | X | X | X |  |  |  |  |  |  |  |  |  | Akko - Ashdod |
| Tripterygion tripteronotus | X | X | X |  | X |  |  |  |  |  |  |  | RoshHaNikra - Ashdod |
| Umbrina cirrosa |  |  |  |  |  | X | X |  |  |  |  |  | Akko - Zikim |
| Uranoscopus scaber |  |  |  | X |  |  | X |  |  |  |  | X | Akko - Yaffo |
| Xyrichthys novacula |  |  |  | X |  |  | X |  |  |  |  | X | Nahariya - Yaffo |
| Zebrus zebrus | X | X | X |  |  | X |  |  |  |  |  |  | RoshHaNikra - Mikhmoret |
| Zeus faber |  |  |  | X |  |  | X |  |  |  |  |  | Akko - Ashdod |

**Table S3.**  Calculation of the value of Israel’s oil and gas reserves using annual average prices from 2012. The crude oil (petroleum) annual average price for 2012 was 404.52 NIS per bbl ([World Bank](http://data.worldbank.org/data-catalog/commodity-price-data)). The natural gas annual average price for 2012 was 399.33 NIS per thousand cubic metres ([International Monetary Fund](http://www.imf.org/external/np/res/commod/index.asp)). The conversion rate was 1 US$ = 3.86 NIS (annual average conversion rate 2012; IMF)

|  | **Reserves**  (Varshavsky 2012) | **Conversion into monetary values**  **NIS billion (US$ million)** |
| --- | --- | --- |
| **Gas reserves** | Proved = 278 Bcm  Contingent = 522 Bcm  Prospective = 680 Bcm | 111.01 (28.76)  208.45 (54.00)  271.54 (70.35) |
| Total | 1,480 Bcm | 591.01 (153.11) |
| **Oil reserves** | Contingent = 230 MMbbl  Prospective = 1,400 MMbbl | 93.04 (24.10)  566.33 (146.72) |
| Total | 1,630 MMbbl | 659.37 (170.82) |
| **Total** |  | **1,250.28 (323.93)** |

*MMbbl = one million barrels; Bcm = billion cubic meters

**Table S4.** Nine species that had the 5% conservation zone (no-take) constraint removed in order for the planning scenario to reach biodiversity targets. The spatial distribution of these species overlapped with the opportunity cost layer for hydrocarbon extraction.

| Nine Species |
| --- |
| [*Echiodon dentatus*](http://species-identification.org/species.php?species_group=fnam&id=1733) |
| *Enchelycore anatina* |
| *Dasyatis chrysonota* |
| *Parablennius incognitus* |
| *Parablennius rouxi* |
| [*Argyrosomus regius*](http://en.wikipedia.org/wiki/Argyrosomus_regius) |
| [*Auxis rochei*](http://www.fao.org/fishery/species/2492) |
| *Raja montagui* |
| *Tursiops truncatus* |

**Figure S1.** Species richness of 153 native fish species compiled from available studies and the Hebrew University of Jerusalem Fish Collection (for detailed information see Table S1).

**Figure S2.** Fishing effort from trawlers and purse seiners in Israel’s territorial waters of the Mediterranean Sea.

**Figure S3.** Fishing effort from long liners and entangling nets in Israel’s territorial waters of the Mediterranean Sea
