# Supplementary material for: The Crowded Sea: Incorporating Multiple Marine Activities in Conservation Plans Can Significantly Alter Spatial Priorities
Source: PLoS One. 2014 Aug 7;9(8):e104489. doi: 10.1371/journal.pone.0104489 (PMC4125186; doi:10.1371/journal.pone.0104489)
Supplement: File S2 — Supplementary material text. (DOCX) [file pone.0104489.s002.docx]

**File S2. Supplementary Material**

**Description of each planning scenario**

The first scenario “Simple Planning” uses Marxan which aims to maximise conservation objectives for a minimal cost. We defined the cost in this study as the opportunity cost of the commercial fishing sector if an area is turned into a marine reserve. Thus, our aim is to minimise the impact to commercial fishermen and at the same time reach our biodiversity targets (described above). We further extend this scenario “Simple Planning A”, to also incorporate the opportunity cost of offshore oil and gas extraction as quantified above, which we refer to as “Simple Planning B”.

For three planning scenarios we used a zoning tool, Marxan with Zones. This tool is an extension of Marxan, where multiple objectives are minimised to produce a compact system of marine zones. We ran Marxan with Zones using defined zones (see Table 1). Marxan with Zones enable us to meet our biodiversity targets within multiple zones. We set our “Conservation Zone” (no-take zone) to include at least 10% of the total species distribution. Our second scenario is “Basic Zoning” which only uses three zones; Conservation Zone; Benthic Protection Zone and Economic Zone.

The third scenario, Intermediate Zoning scenario, uses all four zones (Table 1) and tests three possible values for the zone effectiveness of the “Exploration Zone”. Zone effectiveness is the percentage of protection a given zone provides for each conservation feature (Watts et al. 2009). These values are difficult to obtain as quantifying the impact of different activities on numerous species and habitats is very difficult. Thus, previous studies (Mills et al. 2011) have used expert opinion to obtain zone effectiveness measurements for conservation features. Due to the difficulty of quantifying the impact of hydrocarbon exploration on the multiple species in the study and the absence of data available on this topic we apply protection levels of A) 25%, B) 50% and C) 75% for the Exploration Zone. These percentages try to represent different hypotheses, one that oil and gas exploration infrastructure yields benefits e.g. artificial reef structures that can increase biodiversity and can protect species, and another that suggests there are many threats e.g. oil spills, sound pollution, light pollution and destruction of benthic structures and organisms when platform installation occurs. For the “Economic Zone” (general usage zone) we assume it has no protection and thus a zone effectiveness of 0% for each feature, the “Conservation Zone” an effectiveness of 100% protection for each features, and for the “Benthic Protection Zone” we used species vulnerability values from expert surveys extracted from published literature (<http://www.fishbase.org/>; Cheung et al. 2005; Donlan et al. 2010; Coll et al. 2010), and for geomorphologic structures, we set a value of 50% due to the unknown zone effectiveness on these formations.

The fourth scenario, Complex Zoning, assumes that all leased and licensed hydrocarbon areas in the territorial waters should be avoided when planning a marine reserve to prevent future incompatible objectives with this industry. Therefore we assume that this prospective hydrocarbon area can only be allocated to either the Exploration Zone or the Economic Zone. In this scenario we also incorporate shipping lanes, desalination plants and pipelines (see Table 1 for a list of activities and their placement in specific zones).

**Method of deriving biodiversity features data**

*Fishes:* We removed fish species that had less than two sightings in the past ten years on the basis of expert opinion that these are species are rare and their distribution is unknown. Removed species included: *Eutrigla gurnardus, Microlipophrys nigriceps, Remora remora, Rhinobatus cemiculus, Scomber scombrus, Sprattus* sprattus. Thus, resulting in a total of 153 fish species. It is important to note that none of the eight studies we derived our information from have been conducted at depths greater than 200m. Thus, we have little understanding of the number of species present in deeper territorial waters. However, it is expected that species richness and abundance declines as the continental shelf ends and slopes towards the depths of the ocean floor (Morantal et al. 1998; Kallianiotis et al. 2000; D’Onghia et al. 2004; Tecchio et al. 2011). Experts that were used in this study to verify distribution and ranges included; Dr. Golani, Dr. Goren, Dr. Rilov, Dr. Edelist and Dr. Brokovich.

*Cetaceans:* We only included the Common Bottlenose Dolphin (*Tursiops truncatus*) in this study. While other regular mammal species that visit Israel’s waters include the: Striped Dolphin, Common Dolphin, Risso’s Dolphin, Rough-toothed Dolphin and Cuvier’s Beaked Whale (Kerem et al. 2010) not enough observational data exists to determine priority habitats for these species. Surveys for the Common Bottlenose Dolphin were recorded in Scheinin (2010) along with extra sightings from Israel Marine Mammal Research & Assistance Centre (IMMRAC) between the years 2003 - 2011. From these sources sightings of the Common Bottlenose Dolphin have been recorded throughout Israel’s territorial waters. A sampling effort analysis on the available data provided by A. Scheinin was performed by Israel’s Nature and Parks Authority and the three core feeding and foraging areas were identified from this analysis.

**Methods for calculating opportunity cost of commercial fishers in Israel.**

***Opportunity cost of commercial fishers***

We developed spatial fishing effort maps for all four fishing gears used in Israel; entangling nets, longliners, purse seiners and trawlers (see Fig. S2; S3). Effort maps were compiled by expert opinion, which involved seventeen experienced fishers, two fisheries rangers, six marine scientists including experts from the department of Fisheries and Agriculture. Using these effort maps we further created surrogate cost layers by overlying the 2009 annual revenue reported by Edelist et al. (2013) for each fishing gear type. For the combined fishing effort for Israel’s Mediterranean territorial waters (see Fig. 2b).

*Entangling nets:*

The maximum depth that entangling nets are used in Israel’s waters is ~50 m (confirmed by 15 entangling net fishers in Israel). Using bathymetry data (Amante 2009) we cropped our study area from the coast line to the 50m contour line. We distributed the 2009 entangling net captured biomass (615.6 ton) which is valued at US$ 3.53 million (Edelist et al. 2013) across the designated fishing area. Specifically, we weighted our cost layer by the number of entangling net fishing boats at each port along the coastline of Israel (data provided by Department of Fisheries & Aquaculture 2010) and assume that effort decreases exponentially with distance from port (see Mazor et al. 2013). Thus, each planning unit (1 km^2^) *pu* represents the total annual revenue that is extracted from this area using entangling nets and can be defined by:

$pu revenue= \frac{effort * pu area}{\sum of all pu (effort * pu area)}*revenue$*(US$)*

$$effort = \sum(number of fishing boats at port * exp (-0.01 * distance to port))$$

*Longliners:*

The majority (~90% of fishermen) of longliners are used in Israel’s waters at depths less than ~50 m (confirmed by 6 long line fishers in Israel). While there are a few long liners that operate beyond this depth, for this study we have set a maximum depth of 50 metres. Using bathymetry data (Amante 2009) we cropped our study area from the coast line to the 50m contour line, remaining with 1,784 planning units. We used the 2009 biomass (130 ton) which is valued at US$ 1.56 million (Edelist et al. 2013). As a surrogate measure to spatially represent the revenue of long liners within Israel’s waters we weighted each planning unit with revenue values that decrease exponentially with distance from ports (weighted by the number of longliner fishing vessels at each port; see Equation 1 & 2)) and by distance to nearest rocky habitat (kukar ridges; The Israel Nature and Parks Authority 2012) which are targeted in long line fishing. As longliner fishing efforts are concentrated mostly on rocky habitats we set a weighting so the revenue in each planning unit was:

*pu revenue = (effort on rocky habitats * 0.75) + (effort from ports * 0.25)*

*Purse Seiners:*

There are two distinct areas for purse seiners in Israel. One is concentrated in the north in the Haifa Bay and the second area is in the south between Ashdod to Tel-Aviv at a depth of ~10 – 50m (concentration of effort around a pipeline that is at a depth of ~30m). Here we weighted planning units by the % of effort as derived from expert opinions (6 purse seine fishers) and spread the annual revenue of purse seiners ($US 1.38 million; Edelist et al. 2013) over these weighted planning units.

*Trawlers*:

Trawling lines recorded by on-boat GPS devices were obtained by Edelist (2013) between the years 2009 – 2011. We combined this trawling data with extra trawling data mapped by the Ministry of fisheries (Israel Department of Fisheries 2012). Using this combined data we used the Kernel Density tool in ArcGIS (ESRI 2010) to calculate a magnitude per planning unit from the trawl line (polylines) features. The annual 2009 revenue value for trawling (US$ 6.67 million; Edelist et al. 2013) was overlaid and weighted to reflect this effort distribution.

**Description of economic activities and threats in Israel’s Mediterranean waters.**

*Aquaculture:*

There is currently only one approved aquaculture farm in Israel’s Mediterranean waters off the coast of Ashdod. Currently the only species farmed is Sea bream *Sparus aurata.* The existing aquaculture facilities comprise an area of ~14 km^2^ (Department of Fisheries & Aquaculture 2013). This data was provided by the Ministry of Fisheries and Aquaculture, State of Israel Ministry of Agriculture & Rural Development (Department of Fisheries & Aquaculture 2013). These areas are locked into the “Economic Zone”.

*Current protected areas:* There is currently one marine protected area at the northern border of Israel “Rosh Hanikra” which is controlled and monitored by The Israel Nature and Parks Authority. This is an area of 11.4 km^2^. This spatial GIS data were provided by The Israel Nature and Parks Authority (2012).

*Desalination plants:*

Spatial data were provided by the Israel Ministry of Interior from the National Master plan of Israel (Tama 34b).

*Diving:*

There are 47 dive sites in Israel’s Mediterranean Sea territorial waters. Of these, 34 are shipwreck sites and 13 are natural dive sites. Data was provided by Feder (2012).

*Exploration safety zones:*

Active hydrocarbon drilling platforms are required to implement a 500 m radius around the platform as a safety area following the International Maritime Organization Safety Zone Resolution A.671 (1989; IMO 2013). This area prevents access of these waters to all other activities such as diving, fishing and scientific research. In the territorial waters of Israel only one active drilling area around the Mari B platform has these restrictions. We have locked out this whole planning unit (1 km^2^) from our analysis as no activity is permitted inside this area.

*Military areas (Fire Zones):*

There are several military areas or divisions within the Mediterranean Sea of Israel (see “Fires Zones” in Figure 3). Most of these allow access to fishermen however there are two areas (see “Military areas” in Figure 3) that restrict entry 1) area near Atilt, and 2) a buffer zone adjacent to the Gaza border (~0.5 km). These areas perhaps act as de-facto marine reserve areas where little exploitation of resources is occurring. A comparative study was done (Sonin, 2008) and it was found that these military controlled areas harbour fish species with greater biomass and diversity. Spatial GIS data for military areas were provided by The Israel Nature and Parks Authority (2012).

*Shipping lanes:* Here we geo-referenced and digitised shipping lanes using ArcGIS 10 (ESRI, 2010) from a map provided by the Society for the Protection of Nature in Israel (SPNI) Open Landscape Institute (OLI).

*Pipelines*

Data of offshore oil and gas pipe lines were provided by the Israel Ministry of Interior from the National Master plan of Israel (Tama 37). This data included; 1) existing pipelines and 2) planned pipelines for the transmission of natural gas.

**References:**

Amante C, Eakins BW (2009) ETOPO1 1 Arc-Minute Global Relief Model: Procedures, Data Sources and Analysis. Colorado, USA: NOAA Technical Memorandum NESDIS NGDC-24. Available: <http://www.ngdc.noaa.gov/mgg/global/> Accessed 2013 March 24.

Cheung, W. W. L., T. J. Pitcher, and D. Pauly. 2005. A fuzzy logic expert system to estimate intrinsic extinction vulnerabilities of marine ﬁshes to ﬁshing. Biological conservation **124**: 97–111.

Coll, M., et al. 2010. The Biodiversity of the Mediterranean Sea: Estimates, Patterns, and Threats. PLoS ONE **5**: e11842. doi:10.1371/journal.pone.0011842

D’Onghia, G., C. Politou, A. Bozzano, D. Lloris, G. Botllant, L. Sion, and F. Mastrototaro. 2004. Deep-water fish assemblages in the Mediterranean Sea. Scientia Marina **68**: 87-99.

Department of Fisheries and Aquaculture 2013. GIS Aquaculture data, State of Israel Ministry of Agriculture & Rural Development.

Donlan, C. J., D. K. Wingfield, L. B. Crowder, and C. Wilcox. 2010. Using Expert Opinion Surveys to Rank Threats to Endangered Species: A Case Study with Sea Turtles. Conservation Biology **24**:1586-95.

Edelist D (2013) Fishery management and marine invasion in Israel. [PhD thesis]. Haifa, Israel: University of Haifa.

Edelist D, Scheinin A, Sonin O, Shapiro J, Salameh P, Rilov G, Benayahu Y, Schulz D, Zeller D. 2013. Israel: Reconstructed estimates of total fisheries removals in the Mediterranean, 1950–2010. Acta Adriatica 54: 253-263.

ESRI 2010. ArcMap 10.1 Geographical Information System Software. ESRI Inc.

California. Hebrew University of Jerusalem Fish Collection. 2012. The Biological Collections of the Hebrew University of Jerusalem, Givat Ram, Jerusalem.

Feder, M. 2012. Map of dive sites and sunken wrecks in the Mediterranean. The Israel Technical Diving Forum. Available from <http://www.tek-dive.com/>. (accessed September 2012).

IMO. 2013. International Maritime Organization Safety Zone Resolution A.671 (1989) Safety Zone and Safety of Navigation Around Offshore Installations and Structures. Available from <http://www.imo.org/>. (accessed May 2013).

Israel Department of Fisheries, 2012. Trawling routes along Israel’s Mediterranean coast. 1:100000. Israel, Haifa. Israel Ministry of Agriculture and Rural development - Department Of Fisheries and Aquaculture.

Kallianiotis, A., K. Sophronidis, P. Vidoris, and A. Tselepides. 2000. Demersal fish and megafaunal assemblages on the Cretan continental shelf and slope (NE Mediterranean): seasonal variation in species density, biomass and diversity. [Progress in Oceanography](http://www.sciencedirect.com/science/journal/00796611) **46**: 429–455.

Kerem, D., Hadar, N., Goffman, O., Scheinin, A., Kent, R., Boisseau, O., & Schattner, U. (2012). Update on the Cetacean Fauna of the Mediterranean Levantine Basin. *Open Marine Biology Journal*, *6*.

Mazor T, Giakoumi S, Kark S, Possingham HP (2013) Large-scale conservation planning in a multinational marine environment: cost matters. Ecological Applications  <http://dx.doi.org/10.1890/13-1249.1>

Mills, M., Jupiter, S. D., Pressey, R. L., Ban, N. C., & Comley, J. (2011). Incorporating Effectiveness of Community‐Based Management in a National Marine Gap Analysis for Fiji. *Conservation biology*, *25*(6), 1155-1164.

Mills, M., S. D. Jupiter, R. L. Pressey, N. C. Ban, and J. Comley. 2011. Incorporating Effectiveness of Community-Based Management in a National Gap Analysis. Conservation Biology **26**: 1155-1164.

Moranta, J., C. Stefanescu, E. Massutí, B. Morales, and D. Lloris. 1998. Fish community structure and depth-related trends on the continental slope of the Balearic Islands (Algerian basin, western Mediterranean). Marine Ecology Progress Series **171**: 247-259.

Scheinin A (2010) The Population of Bottlenose Dolphins (Tursiops truncatus), Bottom Trawl Catch Trends and the Interaction between the Two along the Mediterranean Continental Shelf of Israel Haifa, Israel Haifa University.

Tecchio, S., E., Ramírez-Llodra, F. Sardà, and J. B. Baptista. 2011. Biodiversity of deep-sea demersal megafauna in western and central Mediterranean basins. Scientia Marina. **75**: 341-350.

The Israel Nature and Parks Authority (2012) Data - Ecological Geographical Information Centre. Israel Department of Telecommunication and Information Systems.

The Israel Nature and Parks Authority. 2012. Data - Ecological Geographical Information Centre, Department of Telecommunication and Information Systems.

Watts, M. E., Ball, I. R., Stewart, R. S., Klein, C. J., Wilson, K., Steinback, C., ... & Possingham, H. P. (2009). Marxan with Zones: software for optimal conservation based land-and sea-use zoning. *Environmental Modelling & Software*, *24*(12), 1513-1521.
